# Re-examination of 1- vs. 3-Sets of Resistance Exercise for Pre-spaceflight Muscle Conditioning: A Systematic Review and Meta-Analysis

**DOI:** 10.3389/fphys.2019.00864

**Published:** 2019-07-24

**Authors:** Grant W. Ralston, Lon Kilgore, Frank B. Wyatt, Frédéric Dutheil, Patrick Jaekel, Duncan S. Buchan, Julien S. Baker

**Affiliations:** ^1^Applied Physiology Research Laboratory, School of Science and Sport, Institute of Clinical Exercise and Health Sciences, University of the West of Scotland, Hamilton, Scotland; ^2^Kilgore Academy, Azle, TX, United States; ^3^Department of Athletic Training and Exercise Physiology, Midwestern State University, Wichita Falls, TX, United States; ^4^Université Clermont Auvergne, CNRS, LaPSCo, Physiological and Psychosocial Stress, University Hospital of Clermont–Ferrand, CHU Clermont–Ferrand, Preventive and Occupational Medicine, WittyFit, Clermont–Ferrand, France; ^5^Faculty of Health, School of Exercise Science, Australian Catholic University, Melbourne, VIC, Australia; ^6^Space Medicine Team, European Astronaut Centre (EAC), European Space Agency, Cologne, Germany; ^7^KBR, Cologne, Germany

**Keywords:** resistance training and muscular strength, resistance training and training volume, single vs.multiple-sets, one vs.multiple-sets and muscular strength, one vs.three-sets and muscular strength

## Abstract

**Background:** Recommendations on resistance training (RT) set-volume protocols in preparation for spaceflight muscular strength conditioning remains equivocal. A meta-analysis was performed on the effects of single-set (S), or three-set (M3) RT on muscular strength per exercise for different body segments and joint types (multi-joint and single-joint).

**Methods:** Computerized searches were performed on PubMed, MEDLINE and SPORTDiscus™. Twelve studies were considered appropriate according to pre-set eligibility criteria. Outcomes analyzed were pre-to-post-muscular strength change on; multi-joint and single-joint combined; upper body only; lower body only; multi-joint exercises only; single-joint exercises only.

**Results:** Upper body exercise analysis on combined subjects and untrained subjects only reported greater but not significant strength gains with M3 (ES 0.37; 95% CI 0.09–0.82; *P* = 0.11 and ES 0.35; 95% CI−0.49 to 1.19; *P* = 0.42). Trained only subjects reported superior strength gains with M3 (ES 0.63; 95% CI 0.34–0.92; *P* = <0.0001). Lower body exercise on combined subjects and untrained subjects only reported superior strength gains with M3 (ES 0.35; 95% CI 0.10–0.60; *P* = 0.006 and ES 0.49; 95% CI 0.14–0.83; *P* = 0.005). Trained subjects only observed greater but not significant strength gains with M3 (ES 0.18; 95% CI −0.23 to 0.58; *P* = 0.39). Multi-joint exercise on combined subjects reported greater strength gains with M3 (ES 0.83; 95% CI 0.14–1.51; *P* = 0.02). Trained only subjects reported greater strength gains with M3 (ES 0.52; 95% CI 0.10–0.94; *P* = 0.02). Single-joint exercise on combined subjects and untrained only observed greater strength gains for M3 (ES 0.49; 95% CI 0.26–0.72; *P* = <0.0001 and ES 0.56; 95% CI 0.21–0.91; *P* = 0.002). Trained only subjects reported greater but not significant strength gains with M3 (ES 0.37; 95% CI −0.01 to 0.75; *P* = 0.06).

**Conclusion:** For astronauts in space-flight preparation, the findings suggest that M3 training appears to be preferable over S for developing muscular strength. Nevertheless, depending on the physical conditioning of the crew member or tight pre-flight scheduling, S is still able to provide a positive strength training stimulus.

## Introduction

Recent advances in space technology, space medicine and collaboration among international space agencies, have contributed significantly toward sending humans deeper into interplanetary space. It is predicted that future crewed missions will focused on deeper space transit; however, at present, this has not transpired due to the significant demands placed on the human body. Governments and space agencies, however, are determined to achieve long duration space exploration and for this to be achieved the astronauts in-flight physical conditioning must be optimal for mission functionality. Astronauts, as part of pre-flight preparation, follow appropriate resistance training (RT) protocols that prepare them for microgravity (μG) environments. Currently, astronauts live and work in extreme environments but significant differences between low-earth orbit operations and exploring interplanetary space exist. Astronauts presently perform low-earth orbit operations in extreme environments including μG, confinement, radiation exposure, and social isolation. These extreme conditions significantly alter the physiological demands experienced by International Space Station (ISS) astronauts relative to terrestrial dwelling. Spaceflight poses unique physiological deconditioning and maladaptation due to prolonged exposure to μG, including significant muscle degradation and impaired skeletal functioning (Convertino, 1990; Stein, 2013; Bloomberg et al., 2016).

Exposure to a μG environment has been shown to have significant adverse effects on skeletal muscle tissue including changes in expression of structural, metabolic, and contractile proteins that adjust the function of tissue (LeBlanc et al., 1996; Fitts et al., 2001; Trappe et al., 2001; Adams et al., 2003; Carpenter et al., 2010). A reduction in muscle strength also leads to a reduction in applied mechanical forces to bones that may intensify the loss of bone mineral content that occurs due to the lack of ground reaction forces in a μG environment. As a result, astronauts will be physically weaker with bones more fragile when they land. These extreme effects of μG on muscle tissue in humans raised concerns by the National Aeronautics and Space Administration (NASA) about the structural and functional deconditioning in muscles that led to astronauts having; (1) loss of strength to perform emergency egress when landing in partial μG and; (2) the inability to perform and endure occupational activities in μG which vary in the magnitude of work-related loading and intensity (Adams et al., 2003). Widrick et al. (1999) suggested that exposure to two and a half weeks of μG led to an overall eight per cent reduction in fiber diameter or up to 15% in the cross-sectional area of slow twitch muscle fibers of the human soleus. LeBlanc et al. (2000) reported that during a 17 day mission significant post-flight changes occurred in muscle volume of between three-to-ten per cent in all muscle regions except hamstrings compared to baseline. LeBlanc and colleagues also observed significant decreases in muscle volume of between 5 and 17% in all muscle groups except the neck during Mir missions of 16–28 week durations. In addition, Trappe et al. (2009) reported losses of muscle strength and approximately two per cent muscle volume per month and five per cent in peak muscle power per month. Similarly, Gopalakrishnan et al. (2010) stated that up to four per cent loss of strength at the knee per month and a loss of approximately three per cent in elbow strength per month. This reduction in muscle activity during spaceflight compromises muscle mass and strength and could have significant consequences related to the success of long duration space exploration.

These decremental changes have driven the pursuit of adequate pre-spaceflight physical training protocols and suitable countermeasures, which has included electrical stimulation, artificial gravity, nutritional therapy, pharmacologic, and various forms of exercise interventions (Lang et al., 2017). Convertino and Sandler (1995) state that physical exercise is central to inhibit unloading-induced remodeling of the muscular and skeletal system. However, sustaining muscle and skeletal bone health remains a significant obstacle in human space exploration. Current pre-flight prescription of RT is primarily established from evidence-driven terrestrial RT and experience gained during previous missions. This has led to disparities in the physical conditioning of astronauts as no such established exercise prescription has been employed that would sustain in-flight muscle strength and functioning. Unfortunately, there does not appear to be a collectively accepted method regarding pre-flight RT prescription that all space agencies adhere too in preparation for space transit. With the daily set-volume, resistance loading, exercise type, and training frequency vary from the space agency to space agency.

The European Space Agency strategy for astronaut's pre-flight preparation focus on individualized training approaches that incorporate three stages (Kozlovskaya et al., 1995); (1) adaptation phase that acquaints individuals with ISS exercise hardware; (2) main phase that counteracts physiological adaptation to μG and; (3) preparation for re-entry and terrestrial landing. The RT prescription comprises of both multi-joint and single-joint exercises (squats, deadlifts, bench press, crunches, and heel raises) altering from training session to training session (Hackney et al., 2015). The Japan Aerospace Exploration Agency implements a pre-flight programme that consists of individualized programmes that are related to the anticipated mission tasks that crew members would perform (Loehr et al., 2015). The Canadian Space Agency uses a three-block approach with each stage lasting 4 weeks with set-volume for strength between two-to-five sets (Loehr et al., 2015). NASA implement increased set-volume (MS) with astronauts performing both concentric and eccentric actions that are prescribed by the American College of Sports Medicine (Garber et al., 2011).

Garber et al. (2011) constructed the position statement that provides direction on the prescription of exercise for apparently healthy adults. In the 2011 position statement, multiple-sets are cited for experienced trainees and competitive athletes that are comparable to astronauts' fitness status at the end of pre-flight conditioning. However, Stein (2013) argues that astronauts at their physical peak may have more to lose during in-flight unloading because of μG. Matsumoto et al. (2011) reported that astronauts who performed walking as part of the pre-flight protocol lost less body weight than those that performed intense exercise protocols. It could be debated that if astronaut's pre-flight physical conditioning is in an over-compensated state, they may experience more significant weight loss during spaceflight that may be detrimental. Consequentially, the set-volume training dose needed for astronauts to be in optimal condition requires further investigation. Daily RT set-volume has been an often-contested issue, established from different recommendations that support MS programming. However, in preparation for spaceflight with μG environments, it is perhaps more advantageous to implement S programming in which to develop functional strength that does not facilitate the same level of in-flight deconditioning and weight loss.

Published RT meta-analytical evidence is equivocal on what set-volume elicits superior strength improvements, with disparity existing in the recommendations (Table 1). Several meta-analytical studies have been performed that support the use of multiple-sets (MS) programmes compared to single-set (S) per exercise on untrained and trained subjects (Rhea et al., 2002b, 2003; Peterson et al., 2004; Wolfe et al., 2004; Krieger, 2009; Fröhlich et al., 2010). However, due to the absence of available studies, most meta-analytical evidence is drawn from S and MS (two-eight-sets per exercise) that does not fully quantify a dose-response relationship. Several meta-analyses that support increased set-volume (Rhea et al., 2002b, 2003; Peterson et al., 2004; Wolfe et al., 2004) include small ESs that potentially drifted toward greater set-volume. For example, Wolfe et al. (2004) inferred that athletes should perform eight-sets per muscle group to develop strength. This was established from only six effect sizes (ES) and data obtained came from one study and any conclusions derived concerning the direct impact of eight-sets compared to any other number would be unreliable. Besides, none have provided a specific set number for strength development and have pooled findings from studies that have combined different exercise types to generate ES. This, unfortunately, produces issues with daily RT set-volume recommendations, as most meta-analytical evidence have pooled data from studies that have combined exercise types (multi-joint and single-joint exercises) from different population groups (untrained and trained) utilizing a broad age ranges (18–65).

**Table 1:** Summary of previous meta-analyses on set-volume and strength development.

**References**	**Study objective**	**Exercise type**	**Summary findings**
Rhea et al., 2003	Identify a dose-response relationship for intensity, frequency, and volume of training	MJ and SJ comb	Untrained and trained subjects should perform four-sets per muscle group.
Peterson et al., 2004		MJ and SJ comb	Athletes should perform eight-sets per muscle group for athletes.
Peterson et al., 2005	Review of recent evidence on strength development research	MJ and SJ comb	Untrained subjects should perform four-sets per muscle group. Trained subjects should perform eight-sets per muscle group.
Wolfe et al., 2004	Examination of single-set vs. multiple-set on muscle strength	MJ and SJ comb	MS (two-five sets) elicit superior strength gains for trained subjects. Untrained subjects should perform S initially.
Krieger, 2009	Comparison of the effects S-vs.-MS per exercise have on strength	MJ and SJ comb	Maximal strength gains are elicited with two-three-sets per exercise than S, in both untrained and untrained subjects.
Fröhlich et al., 2010	Comparison of the effects of S-vs.-MS for increasing maximal strength levels	MJ and SJ comb	S regimes are equivalent to MS training for increasing strength in the initial period. MS training is superior overextended periods.

*N, number; MJ, multi-joint; SJ, single-joint; comb, combined; MS, multiple-sets; S, single-joint*.

Although meta-analyses regarding the effects of S vs. MS have been published (Rhea et al., 2002b, 2003; Peterson et al., 2004; Wolfe et al., 2004; Krieger, 2009; Fröhlich et al., 2010), with support given for the application of MS to develop strength or muscular hypertrophy. Disagreement remains regarding the need to perform additional sets for increasing muscular strength. Published critical reviews (Smith and Bruce-Low, 2004; Winett, 2004; Otto and Carpinelli, 2006; Carpinelli, 2012; Fisher, 2012), have examined the validity of published meta-analyses on set-volume, concluding that reported data do not fully support a dose-response relationship between the additional number of sets and strength gains. These reviews identified confounding factors including the presence of low-quality studies, variations in subject characteristics and inconsistencies in experimental designs that generate spurious inferences regarding muscular strength increases.

Currently, no meta-analytical evidence is available that examines the effect of daily set-volume on body segmentations (upper or lower body) or specific joint types (MJ and SJ) on muscle strength change. In the context of pre-flight RT, it is critical that the magnitude of daily RT set-volume is examined to prepare astronauts for space transit. The purpose of this review and meta-analysis, therefore, was four-fold: (1) to re-examine the effects of RT volume (S or M3) of ST on muscular strength per exercise; (2) to determine if specific set-volume (S vs. M3) produce different strength gains when multi-joint exercises are compared with single-joint exercises; (3) to investigate if the magnitude of strength gain differs between multi-joint and single-joint exercises by population group (trained vs. untrained) and body segmentations (upper vs. lower body). The final objective; (4) is to provide a perspective on developing muscular strength that provides recommendations on daily RT set-volume for pre-flight strength development. Based on previous evidence (Rhea et al., 2002b, 2003; Peterson et al., 2004; Wolfe et al., 2004; Krieger, 2009), we hypothesized that there would be superior pre-to-post-training strength gains with M3 RT compared to S.

## Methods

### Literature Search

This meta-analysis was performed using the recommendations and criteria defined in the Preferred Reporting Items for Systematic Reviews and Meta-Analyses (PRISMA) statement (Liberati et al., 2009). Computer-aided searches were conducted using the following databases: MEDLINE (PubMed), SWETSWISE, EMBASE, and SPORTDiscus^TM^. The period of search history assessed was inclusive to August 2018. An extensive manual search and cross-referencing of journals, reference lists, was also performed with citations and abstracts from studies published in foreign language journals and scientific conferences were excluded. Descriptive terms and keywords that were used to retrieve studies included: “resistance training and muscular strength,” “resistance training and training volume,” “single vs. multiple-sets,” and “one vs. multiple-sets and muscular strength.” Boolean operators, including AND, OR, and NOT, were used to focus literature searches with literature searches reduced to studies involving humans only.

As a result of systematic computerized database searches, journals were retrieved from 1960 to August 2014 in where S vs. M3 were examined, from different population demographics (trained, untrained, male, and female subjects). After preliminary literature searching, reference lists of articles were screened for additional studies of relevance on muscular strength development. During the first selection round, appropriate study titles were screened for relevance with the inclusion of either resistance training or training volume. In the second selection round, GR, LK, and DB read the abstracts and then selected the article if resistance training for muscle strength was evaluated before and after a minimum RT intervention period of 4-weeks. This minimum time course was chosen due to reports of muscular adaptations in response to RT (Stock et al., 2016). In the third selection round, full articles were read.

### Eligibility Criteria

Studies were deemed eligible in this review if they met the following conditions; (a) human subjects free from chronic disease, muscular, or orthopedic injuries, or physical limitations; (b) trained and untrained adult male or female subjects between 18 and 45 years; (c) subject's descriptive characteristics included in the report (height, weight, training status, and training experience); (d) subjects training at least one primary muscle group-pectoralis major, deltoids (anterior, lateral, posterior); bicep brachii, or tricep brachii; latissimus dorsi; quadriceps (vastus medialis, vastus intermedius, vastus lateralis, rectus femoris); hamstrings (bicep femoris, semitendinosus, semimembranosus; (e) at least one performed pre-to-post measure of muscular strength; (f) studies that compared S vs. M3 performing resistance exercise only (active control group); (g) training protocols lasting a minimum of 4-weeks; (h) and appropriate information to calculate training ES. This meta-analysis included both randomized trials (RAN) and randomized control trials (RCTs) that observed the intervention treatments using stratified resistance exercises with S vs. M3. RAN allocation ensures no systematic variances between the intervention groups; however, no control group may influence the assessment of outcomes (Schünemann et al., 2013). RCTs are a more specific method for defining a cause-effect relationship between treatments and outcomes.

### Search Strategy

Titles and abstracts of retrieved journal articles were independently evaluated for content relevance by three reviewers (GR, LK, and DB). Abstracts that contained the necessary information regarding the pre-set inclusion and exclusion criteria were retrieved and independently evaluated for full-text eligibility. Potential studies that did not have descriptive data tables but presented pre- to post-primary strength data in the form of figures resulted in extraction using WebPlot-Digitiser (Web Plot Digitiser V.3.11. Texas, USA: Ankit Rohatgi, 2017). Where differences between reviewers (GR, LK, and DB) occurred then additional dialogue and agreements were made by consensus. Ten randomly selected studies underwent *post-hoc* reassessment with the extracted results compared. For each reviewer coder drift was set at <10% in all cases, and inter-rater (GR and DB) reliability was >95%. Studies were read and individually coded for the following variables; (1) subject's descriptive characteristics, including age, training experience, and sample size; (2) programme characteristics including training frequency, number of sets performed per exercise, the number of reps performed per exercise; (3) measurement of pre-post-strength outcome(s) and; (4) treatment effects of mean (M) and SD values of changes in pre- and post-strength outcomes for RT intervention and control groups.

### Assessment of Methodological Quality of Studies

Internal validity of retrieved studies was evaluated using the Physiotherapy Evidence Database (PEDro) scale. The PEDro scale (Verhagen, 1998; Maher et al., 2003) has 11 measures, with a maximum score of ten. However, a maximum score from the PEDro scale, in this case, was eight, as the therapists, assessors and technicians conducting the interventions cannot be blinded. Studies were included in this analysis if they had a PEDro score of ≥ four, as this was considered as having acceptable internal validity. Methodological quality was independently assessed by reviewers (GR, LK, and DB). Variances of judgement concerning the scoring of the journal articles were agreed between reviewers through consensus.

### Calculation of Effect Size

Descriptive statistics were calculated to describe and summarize the results of the systematic review process. Data of individual study characteristics were entered into a spreadsheet (Microsoft, Redmond, WA, USA) to compare pre-post-strength outcomes of each study for coding, review and data reference. Descriptive statistics containing sample size (n), mean (M) and SD were extracted from each study. This provided data for the mean differences in pre- to post-intervention between groups (e.g., S and M3) on several strength outcomes. Muscular strength was deemed a continuous data variable; therefore, the standardized mean difference (SMD) with 95% confidence intervals (95% CI) were used to establish the ES measures. For each strength outcome variable, a SD score was calculated by using Cohen's d index of a single ES (di = [M1–M2]/SDpi) (Cohen, 1998), where d = ES, i = individual study, M1 = pre-intervention mean, M2 = post-intervention mean, and SDp = pooled standard deviation. The SD was calculated by summing the extracted pre-intervention and post-intervention SDs and dividing by two. If the standard error of measurement (SEM) of the mean was specified, the SD was calculated using the formula (SD = SEM^*^square root of N) (Howell, 2012). Separate ES was weighted to account for individual sample sizes. If a study reported, exact *P*-values for a change of strength, the SD of change was calculated. Studies that did not report, exact *P*-values, the SD of change was calculated using the pre- and post-intervention SDs. Due to diverse population demographics and methods with the included studies, a random-effects inverse variance (IV) using the DerSimonian-Laird method (DerSimonian and Laird, 1986) was applied with the effects measure of SMD. If a study had numerous time-periods, only the pre- to post-intervention strength outcomes were extracted and entered for analysis. The data was then used to compute ES estimates and CI. For each strength measure, an ES was calculated as the pre- to post-intervention change, divided by the pre-intervention SD (Morris and DeShon, 2002).

Meta-Essentials (Suurmond et al., 2017) was initially used to input pre-post-strength outcome data with each row denoted as an individual ES for a treatment group. If treatment groups had multiple ES, then each ES was coded in a separate row. This aided with the computation of ES, SEM, and study size to allocate appropriate weight to each study, and estimate a study effect. To determine the significance of the ES, the chi-square (Chi^2^) test was performed in each model used. For the statistical analyses, Review Manager (RevMan) version 5.3.5 was used to calculate the difference in SD of post-intervention strength outcomes and the generation of forest plots. Data needed were either; (1) means and SDs (pre- and post-strength change); (2) CI data for pre- to post-strength change for each treatment group (3) *P-*values for pre- to post-strength difference for each treatment group, or if only the level of significance was available, and; (4) default *P*-values (e.g., *P* ≤ 0.05 becomes *P* ≤ 0.49, *P* ≤ 0.01 becomes *P* ≤ 0.0099, and *P* ≥ not significant becomes *P* ≥ 0.05).

The random-effects model was implemented to allow for variability between the studies due to high heterogeneity. A random-effects model conceptualized the existing series of studies under investigation to be a random sample selected from a larger population of studies. In the random-effects model meta-analysis, there are two sources of variability; (1) variability of the effect parameters, and; (2) sampling variability of experimental units (i.e., subjects) into studies. If individual parameter estimates of each study lead to high levels of heterogeneity, the random-effects analysis considers the “*true variance*” (or the remaining unmeasured random-effects between studies) in addition to the modeled between-study variances and sampling error typically assumed in fixed-effects models. It should be highlighted that the random-effects model typically gives less specific estimates and larger CIs.

### Heterogeneity and Risk of Bias

To evaluate heterogeneity between studies, the *I*-squared (*I*^2^) index test and Cochran Q (Q) heterogeneity statistic were applied. The *I*^2^ test was used to assess the degree of heterogeneity for each outcome, with an *I*^2^ > 50% applied to indicate heterogeneity. Non-significance signifies that the results of the different studies were similar (*P* ≥ 0.05) and *P* < 0.05 denotes a statistically significant effect. The Q statistic uses the sum of squared deviations of each estimate resulting from the pooled estimate and weights the contribution of each study. The Q heterogeneity test was applied to evaluate heterogeneity prior to estimating tau-square (Tau^2^), then *I*^2^ and Tau^2^ statistics were calculated. Comparing the Q statistic with an X^2^ distribution with k^−1^ degrees of freedom (where k denotes the number of included studies) allowed *P*-values to be attained. All analyses were conducted at the 95% confidence level. The ES of ≤ 0.2, ≤ 0.5, ≤ 0.8, and ≥0.8 were considered trivial, small, moderate and large, respectively (Cochran, 1954).

For the assessment and evaluation of publication bias, the use of funnel plot assessments with Duval and Tweedie's (2000) trim and fill correction was applied. The purpose of the “*trim and fill*” was to identify and correct for funnel plot asymmetry ascending from publication bias. This method is to; (1) remove the smaller studies causing funnel plot asymmetry; (2) apply the trimmed funnel plot to estimate the true “*centre*” of the funnel, then; (3) replace the removed or omitted studies around the center. Forest plots were produced to display the study-specific ES and the corresponding CI. All forest plots generated were visually examined against its standard error (SE) to account for publication bias also known as the “*file drawer problem*.” This refers to the influence of the results of a study that introduces bias into the scientific literature by selective publication, primarily by the propensity to publish positive results but not to publish negative results (Scargle, 2000).

Separate subgroup analysis on ES was performed with the resulting moderators, including; (1) single-joint or multi-joint resistance exercise on 1RM strength gains (trained only subjects); (2) single-joint or multi-joint resistance exercise on 1RM strength gains (untrained only subjects). In the subgroup analysis, mean differences in ES were computed for each study to produce a study-level ES for the difference between S and M3 allowing for the generation of forest plots. Sensitivity analysis was performed, by identifying any studies that were highly influential which may bias the analysis. This was achieved for each model by eliminating one study at a time and then inspecting the set-volume predictor. Influential studies were removed if they caused a significant change in the magnitude of the coefficient or change from significant (*P* ≤ 0.10) to non-significant (*P* ≥ 0.10) or vice versa.

## Results

**Figure 1:**
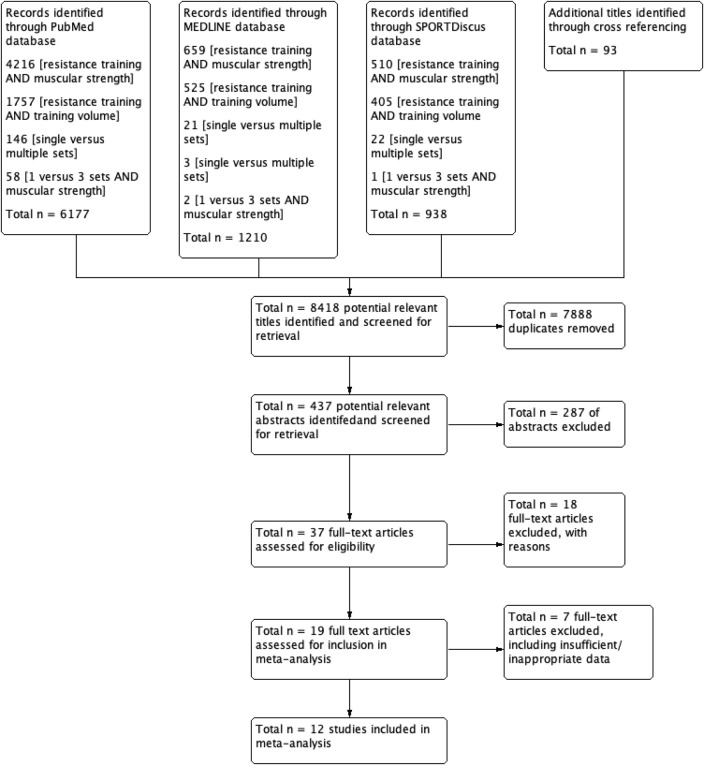
The flow of journal articles through the systematic review process.

The procedure used for systematic literature search and retrieval is displayed in Figure 1 from “*potentially relevant*” to article inclusion. The specific stages of the selection procedure for the meta-analysis are described as a flow diagram (Figure 1).

### Study Selection

The initial examination generated 8,418 related abstracts and citations. Thirty-seven full-text articles were initially deemed to meet the inclusion criteria. A total of 19 potentially relevant journal articles met the pre-set inclusion and exclusion criteria (Table 2) and were further assessed for content applicability. Six studies (Kraemer, 1997; Borst et al., 2001; McBride et al., 2003; Galvão and Taaffe, 2005; Munn et al., 2005; Rønnestad et al., 2007) were rejected prior to data extraction, with Galbraith plot identifying one further article (Starkey et al., 1996) as an outlier and was omitted. Descriptions for the seven studies that were excluded are detailed in Table 3.

**Table 2:** Inclusion and exclusion criteria.

**Inclusion criteria**	**Exclusion criteria**
One or more muscle groups used duration intervention and appropriate strength assessment (i.e., single-joint exercises, e.g., leg curl)	Small subject sample groups (e.g., *n* < six)
The minimum duration of the training intervention is 4-weeks; preferably longitudinal studies (>12-weeks)	The use of either legal or illegal ergogenic aids or supplementation prior to or during interventions
It would be desirable if there were an appropriate control group included within the research design with subjects randomly assigned to groups	Training order variation throughout the intervention
Training programme supervised throughout the intervention. Ensuring that interventions are of similar order and if applicable inter-set recovery periods standardized for multiple-sets	No quasi RCT or narrative studies/reviews to be included
The warm-up is standardized between treatment groups	Subjects below 18 or above 45 years of age
Appropriate criteria were specified regarding training loading (intensity) and subjects trained to volitional fatigue	Researchers did not report results adequately (pre-to-post-mean and standard deviation)
Subject groups comparing 1- vs. 3-sets per exercise per session	Investigated the effects of nutritional supplements in combination with resistance training
Subjects used resistance training as a means of training	Concurrent aerobic and strength training interventions
Studies published in the English language journals only	

**Table 3:** Characteristics of excluded studies investigating 1-vs. 3-sets.

**References**	**Design**	**Status**	**Sex**	**N**	**Age, y, mean ± SD or range**	**Frequency**	**Duration (wk)**	**Sets**	**Reps**	**Strength outcomes**	**The reasoning for exclusion from the current analysis**
Kraemer, 1997	RAN	T	M	40	20± 2.3	3	12	1/3	8–12	1RM BP, 1RM LP	Excluded due to differences between groups, single circuit group performed forced reps at the end, and multiple circuit group did no forced reps
Borst et al., 2001	CT	U	C	31	37± 7	3	25	1/3	8–12	Sum of 1RM for CP and LExt	Excluded due to inadequate evidence of pre-and post-intervention training means and SDs to calculate an effect size
McBride et al., 2003	RCT	U	C	28	21.52± 1.3	2	12	1/3	6–15	1RM LP; 1RM BC	Excluded due to amount of sets subjects were performing
Galvão and Taaffe, 2005	RAN	U	C	28	65–78	2	20	1/3	8	Maximum isokinetic and isometric KExt strength	Excluded due to subjects age range
Munn et al., 2005	RCT	U	C	115	20.6 ± 6.1	3	6	1/3	6–8	1RM EFlex	Excluded due to the primary aim, which was the effects of contraction speed with one or three-sets at fast or slow speeds
Rønnestad et al., 2007	RAN	U	M	21	26.6± 0.1	3	11	1/3	7–10	1RM lower body (LP, LExt, LC); 1RM upper body (CP, Row, LatP, BC, SP)	Provided the subjects with nutritional supplementation (protein chocolate bar) and energy drinks during each exercise bout
Starkey et al., 1996	RCT	U	C	48	18–50	3	14	1/3	8–12	Maximal isometric KFlex; KExt	Study data identified as an outlier when observed using the Galbraith plot

*N, number; y, years; SD, standard deviation; wk, weeks; Reps, repetitions; RAN, randomly assigned trial; T, trained; M, male; 1RM, 1 repetition maximum; BP, bench press; LP, leg press; CT, control trial; U, untrained; C, male and female subjects combined; CP, chest press; LExt, leg extension; SD, standard deviation; RCT, randomized controlled trial; BC, bicep curl; KExt, knee extensor; EFlex, elbow flexor; LC, leg curl; Row, seated row; LatP, latissimus pull-down; SP, shoulder press; KFlex, knee flexion*.

### Resistance Training Study Characteristics

Following appraisal and sensitivity measures 12 full-text articles (Reid et al., 1987; Kraemer, 1997; Hass et al., 2000; Schlumberger et al., 2001; Rhea et al., 2002a; Paulsen et al., 2003; Humburg et al., 2007; Kelly et al., 2007; Bottaro et al., 2009; Baker et al., 2013; Sooneste et al., 2013; Radaelli et al., 2014) met pre-set inclusion criteria (Table 2). Journal articles included in this analysis had dates ranging from 1987 to 2014. In total, 12 studies provided data on 393 subjects (Table 4) with the both randomized control groups (RCT [*n* = 4]) and random assignment of treatment conditions (RAN [*n* = 8]) experimental designs included.

**Table 4:** Study and subject characteristics 1-vs. 3-sets.

**References**	**Design**	**Status**	**Sex**	**N**	**Age, y, mean ± SD or range**	**Until failure**	**Frequency (sessions per wk)**	**Duration (wk)**	**Sets**	**Reps**	**Training loads (% 1RM)**	**Strength outcomes [strength measurement type]**
Hass et al., 2000	RAN	T	C	42	39.2–40.1	Yes	3	13	1/3	8–12	67–80	[1RM] LExt, LC, CP, OP, BC
Rhea et al., 2002a	RAN	T	M	16	20–22	Yes	3	12	1/3	8–12	67–80	[1RM] BP, LP
Paulsen et al., 2003	RAN	U	M	18	20–30	Yes	3	6	1/3	7	83	[1RM] Sq, KExt, LC, BP, SP, Row, LatP
Kelly et al., 2007	RCT	T	C	40	22.2–25.3	Max effort	2	8	1/3	8	80	[Nm] KExt
Bottaro et al., 2009	RAN	U	M	24	19–25.4	Yes	2	12	1/3	8–12	67–80	[1RM] KExt, EExt
Baker et al., 2013	RAN	T	M	16	18–21	Yes	3	8	1/3	6	85	[1RM and Nm] BP, SP, BC
Sooneste et al., 2013	RAN	U	M	8	22.9–27.1	Yes	2	12	1/3	8	80	[1RM] SPC
Reid et al., 1987	RAN	U	M	34	18–35	Yes	3	8	1/2/3	3–18	63–93	[1RM] EFlex, EExt, KFlex, KExt, SFlex, SExt
Kramer et al., 1997	RAN	T	M	43	20.3 ± 1.9 [SEM]	Yes	3	14	1/3	8–12	67–80	[1RM] Sq
Schlumberger et al., 2001	RCT	T	F	27	20–40	Yes	2	6	1/3	6–9	65–77	[1RM] LExt, BP
Humburg et al., 2007	RCT	U	C	29	23.1–27.1	Yes	3	9	1/3	8–12	67–80	[1RM] BC, LP, BP
Radaelli et al., 2014	RCT	U	M	48	23.5–25.3	Yes	3	26	1/3/5	8–12	67–80	[5RM] BP, LP, LatP, SP
Total/mean ± SD				393			2.7 (± 0.49)	11.2 (± 5.4)		9.0 (± 1.7)	76.5 (± 7.5)	

*N, number; y, years; SD, standard deviation; wk, weeks; Reps, repetitions; % 1RM, percentage of subjects one repetition maximum; RAN, randomly assigned trial; T, trained; C, male and female subjects combined; 1RM, 1 repetition maximum; LExt, leg extension; LC, leg curl; CP, chest press; OP, overhead press; BC, bicep curl; M, male; BP, bench press; LP, leg press; U, untrained; SEM, standard error of measurement; Sq, squat; KExt, knee extension; SP, shoulder press; Row, seated row; LatP, latissimus pull-down; RCT, randomized controlled trial; Nm, peak torque; 5RM, subjects five repetition maximum; EFlex, elbow flexion; KFlex, knee flexion; SFlex, shoulder flexion; SExt, shoulder extension; SPC, seated preacher curl; F, female*.

The mean age of the subjects was 25.2 (± 5.4 years). The training status of subjects included in the 12 studies was untrained (*n* = 6) and trained (*n* = 6). Assigned cohorts consisted of male (*n* = 8 [67%]), female only groups (*n* = 1 [12%]), and mixed-sex studies (*n* = 3 [25%]) which were included in the analysis. The RT period ranged from 6 to 26 weeks (mean = 11.2 [±5.4] weeks), weekly training frequency ranged from 2 to 3 days per week (2.7 [±0.49] per week), and the repetitions used ranged from 3 to 18 repetitions (9.0 [±1.7]) per week. The total number of sets per week ranged from two-to-three-sets (2.7 [±1.7]) for S and six-to-nine-sets for M3 (7.5 [±2.1]) per exercise. Also, training loads ranged from 63 to 90% 1RM (76.7 [±4.1]) with the subject's resistance training characteristics and weekly training volume specified in Table 5.

**Table 5:** Resistance training characteristics and weekly training volume.

**References**	**RT exercises performed**	**Total number of sets per exercise performed weekly**	**Total number of reps performed daily per exercise**	**Total number of reps performed weekly per exercise**	**Total number of reps (sets × reps × frequency × exercise) performed weekly**
Hass et al., 2000	LExt, LC, PullO, ACross, CP, LatR, OP, BC, TriExt	S:3 M3:9	S: 8–12 M3: 8–12	S:24–36 M3:72–108	S = 216–324 M = 648–972
Rhea et al., 2002a	BP, LP. S performed additional exercises BC, LatP, AbC, BExt, Row	S:3 M3:9	S: 8–12 M3: 8–12	S:24–36 M3:72–108	S = 168–252 M3 = 144–216
Paulsen et al., 2003	Sq, KExt, LC, BP, SP, Row, LatP	S:3 M3:9	S: 7 M3: 7	S:21 M3:63	S = 147 M3 = 441
Kelly et al., 2007	KExt	S:2 M3:6	S: 8 M3: 8	S:16 M3:48	S = 16 M = 48
Bottaro et al., 2009	LP, PullO, KFlex, CP, BC, AbC	S:2 M3:6	S: 8–12 M3: 8–12	S:16–24 M3:48–72	S = 96–144 M = 288–432
Baker et al., 2013	BP, IncBP, DumF, BCbar, BCdumb, HammerC, SP, LatR, URow	S:3 M3:9	S: 6 M3: 6	S: 18 M3: 54	S = 162 M = 486
Sooneste et al., 2013	SPC	S:2 M3:6	S: 8 M3: 8	S:16 M3:48	S = 16 M = 48
Reid et al., 1987	LExt, LC, LP, CR, BP, MilPres, LatP, TriExt, BC	S:3 M3:9	S: 8 M3: 8	S:24 M3:72	S = 216 M = 648
Kramer et al., 1997	Sq, PushP, BP, AbC, PullTh, LC, BRow	S:3 M3:9	S: 8–12 M3: 8–12	S:24–36 M3:72–108	S = 168–252 M = 504–756
Schlumberger et al., 2001	LExt, LC, AbC, ShAdd, ShAbd, BP, LatP	S:2 M3:6	S: 6–9 M3: 6–9	S:12–18 M3:36–54	S = 84–126 M = 252–378
Humburg et al., 2007	BC, LP, BP	S:3 M3:9	S: 8–12 M3: 8–12	S:24–36 M3:72–108	S = 72–108 M = 126–324
Radaelli et al., 2014	BP, LP, LatP, LExt, SP, LC, BC, AbC, TriExt	S:3 M3:9	S: 8–12 M3: 8–12	S:24–36 M3:72–108	S = 216–324 M = 648–972

*RT, resistance training; LExt, leg extension; LC, leg curl; PullO, pull-over; ACross, arm cross-over; CP, chest press; LatR, lateral raise; OP, overhead press; BC, bicep curl; TriExt, tricep extension; BP, bench press; S, one-set; M3, three-sets; LP, leg press; LatP, latissimus pull-down; AbC, abdominal curl; BExt, back extension; Row, seated row; Sq, squat; KExt, knee extension; SP, shoulder press; KFlex, knee flexion; IncBP, incline bench press; DumF, dumbbell flye; BCbar, bicep curl with bar; BCdumb, bicep curl with dumbbells; HammerC, hammer curl; URow, upright row; SPC, seated preacher curl; MilPres, military press; PushP, push press; PullTh, pull through; Brow, back row; SHadd, shoulder adduction; ShAbd, shoulder abduction*.

### Sensitivity Analysis

**Figure 2:**
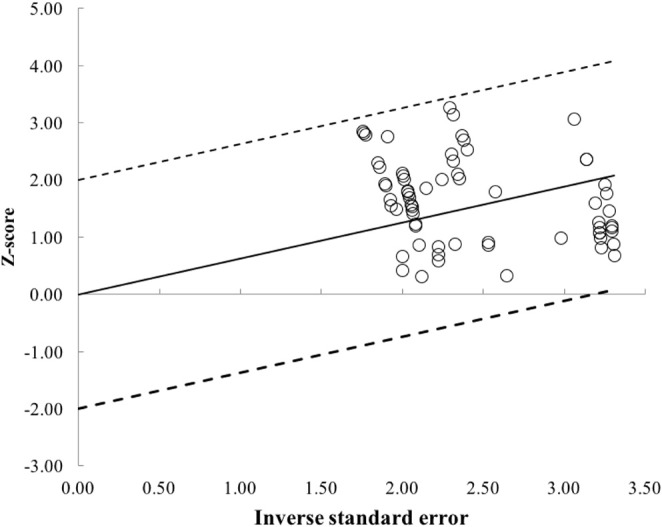
Galbraith plot used to examine study heterogeneity (pre- vs. post-strength change). Each open circle represents one pre- vs. post-study data.

The PEDro scale was based on the Delphi list (Verhagen, 1998) with column 1a not used in the calculation of the scores. Only criterion 2–11 are scored giving a total out of ten. Each column number corresponds to the following criteria on the PEDro scale (Table 6): 1^a^ = eligibility criteria (1a = eligibility criteria specified [1 = yes/0 = no]); 2 = subjects randomly allocated; 3 = allocation was concealed; 4 = groups similar at baseline; 5 = blinded subjects; 6 = therapists blinded; 7 = assessors blinded; 8 = follow-up measures obtained for >85% of subjects; 9 = intention to treat analysis; 10 = between groups statistical comparison; 11 = point measures and measures of variability. The included studies had PEDro scores that ranged from five through to six (Table 6). Though the maximum PEDro score is 11, it is problematic and unrealistic to achieve this total. Realistically the maximum score on PEDro was eight as it is problematic to blind both participants and researchers to an exercise intervention. Consequently, included journal articles had a common area of bias as subjects, therapists or researchers were not blinded. Galbraith plots were used to investigate for study heterogeneity and identification of potential outliers. Examination of Galbraith plots exposed no outliers (Figure 2). To assess for publication bias trim and fill funnel plot were also performed in all comparison models. This was to safeguard for overestimations of the ES of set-volume and strength outcomes in the included studies. The shape of the funnel plot did not expose any evidence of apparent asymmetry.

**Table 6:** Methodological quality of studies based on the PEDro score.

**References**	**PEDro Scale item**											**Total**
	**1^a^**	**2**	**3**	**4**	**5**	**6**	**7**	**8**	**9**	**10**	**11**	
Reid et al., 1987	1	1	0	1	0	0	0	1	1	1	1	6
Kramer et al., 1997	1	1	0	1	0	0	0	1	1	1	1	6
Hass et al., 2000	1	1	0	1	0	0	0	1	1	1	1	6
Schlumberger et al., 2001	1	1	0	1	0	0	0	1	1	1	1	6
Rhea et al., 2002a	1	1	0	0	0	0	0	1	1	1	1	5
Paulsen et al., 2003	1	1	0	1	0	0	0	1	1	1	1	6
Humburg et al., 2007	1	1	0	1	0	0	0	1	1	1	1	6
Kelly et al., 2007	1	1	0	1	0	0	0	1	1	1	0	5
Bottaro et al., 2009	1	1	0	1	0	0	0	1	1	1	1	6
Baker et al., 2013	1	1	0	1	0	0	0	1	1	1	1	6
Sooneste et al., 2013	1	1	0	1	0	0	0	1	1	1	1	6
Radaelli et al., 2014	1	1	0	1	0	0	0	1	1	1	1	6

*PEDro, Physiotherapy Evidence Database. The PEDro scale is based on the Delphi list (Verhagen, 1998). Column 1a not used in the calculation of the scores. Only criterion 2–11 are scored giving a total out of ten. Column numbers correspond to the following criteria on the PEDro scale*:

1a * = eligibility criteria (1a = eligibility criteria specified [1 = yes/0 = no]), 2 = random allocation, 3 = concealed allocation, 4 = groups similar at baseline, 5 = blinded subjects, 6 = blinded therapists, 7 = blinded assessors, 8 = follow-up measures obtained for > 85% of subjects, 9 = intention to treat analysis, 10 = between groups statistical comparison, 11 = point measures and measures of variability*.

### Effects of 1- vs. 3- Sets on Multi-Joint and Single-Joint Exercise

Pre- to post-strength outcomes were assessed via a meta-analytic procedure for all included studies. Subgroup analysis was then performed with multi-joint and single-joint exercises combined into separate subdivision analysis. A random-effects model was incorporated into each strength measure due to the potential of pooled study data generating significant heterogeneity with *I*^2^ used to evaluate heterogeneity.

The pooled mean ES estimates (untrained and trained) of multi-joint and single-joint data (Tables 7, 8) comprised of 70 treatment groups from 12 studies (Reid et al., 1987; Kramer et al., 1997; Hass et al., 2000; Schlumberger et al., 2001; Rhea et al., 2002a; Paulsen et al., 2003; Humburg et al., 2007; Kelly et al., 2007; Bottaro et al., 2009; Baker et al., 2013; Sooneste et al., 2013; Radaelli et al., 2014). The random-effects model exposed a considerable amount of variability between studies. Heterogeneity prior to taking Tau^2^ into consideration (Q heterogeneity test) was: Chi^2^ = 131.56, d.f. = 11, *P* < 0.00001. The heterogeneity statistic *I*^2^ (%) = 92 [interpreted as high, (Higgins et al., 2003)], and the tau^2^ test (between-trials variance) = 0.74. When a random effect analysis was implemented, a large effect was detected for combined multi-joint and single-joint exercises on RT set training volume [mean effect size (ES) 0.93; 95% CI 0.41–1.45]. Pre- to post-intervention strength change was greater with M3 compared to S (ES difference 0.25) with the effect statistically significant (*P* = 0.0005). The mean for S was 0.64 (95% CI 0.44–0.84). The mean ES for M3 was 0.89 (95% CI 0.66–1.12).

**Table 7:** Pre- vs. post-strength analysis of multi-joint exercise.

**References**	***N***	***N* per group**	**Age (y) [range or mean ± SD]**	**Frequency/duration**	**Testing modality**	**Sets (reps)**	**Training loads**	**Weekly sets per exercise**	**Pre- vs. post [mean ± SD]**	**Pre- vs. post % strength change**	**Reported *P*-value (Pre- vs. post)**	**ES**
Hass et al., 2000	42	S:21	39.2–40.1	3 per wk for 13 wks	CP	1 (8–12)	8–12RM	S	1.9 ± 0.6 vs. 2.1 ± 0.5	10.5	≤0.05	0.36
		M3:21			CP	3 (8–12)		M3	2.1 ± 0.7 vs. 2.3 ± 0.6	9.5	≤0.05	0.31
Hass et al., 2000	42	S:21	39.2–40.1	3 per wk for 13 wks	OP	1 (8–12)	8–12RM	S	1.9 ± 0.4 vs. 2.0 ± 0.4	5.3	≤0.05	0.25
		M3:21			OP	3 (8–12)		M3	2.0 ± 0.6 vs. 2.3 ± 0.6	15.0	≤0.05	0.5
Rhea et al., 2002a	16	S:8	19–23	3 per wk for 12 wks	LP	1 (8–12)	8–12RM	S	269.0 ± 16.8 vs. 337.2 ± 69.0	25.4	≤0.05^a^	1.36
		M3:8			LP	3 (8–12)		M3	225.9 ± 25 vs. 343.5 ± 89.9	52.1	≤0.05^a^	1.78
Rhea et al., 2002a	16	S:8	19–23	3 per wk for 12 wks	BP	1 (8–12)	8–12RM	S	64.2 ± 8.9 vs. 76.7 ± 28.0	19.5	≤0.05^a^	0.60
		M3:8			BP	3 (8–12)		M3	66.8 ± 7.3 vs. 85.5 ± 20.8	28	≤0.05^a^	1.20
Paulsen et al., 2003	18	S:10	20–30	3 per wk 6 wks	Sq	1 (7)	7RM	S	129.5 ± 65.1 vs. 147 ± 67.4	13.5	≤0.01^b^	0.26
		M3:8			Sq	3 (7)		M3	122.5 ± 82.0 vs. 149.4 ± 82	22.0	≤0.01^b^/≤0.05^c^	0.33
Paulsen et al., 2003	18	S:10	20–30	3 per wk for 6 wks	BP	1 (7)	7RM	S	74.8 ± 22.1 vs. 82.3 ± 26.3	10	≤0.01^b^	0.31
		M3:8			BP	3 (7)		M3	77.8 ± 32 vs. 85.0 ± 36.5	9.3	≤0.01^b^/≤0.05^c^	0.21
Baker et al., 2013	16	S:8	18–21	3 per wk for 8 wks	BP	1 (6)	6RM	S	659.4 ± 112.7 vs. 776.2 ± 121.5	17.7	≤0.05 one tailed^a^	1.00
		M3:8			BP	3 (6)		M3	671.3 ± 131.3 vs. 789.9 ± 96.0	17.7	≤0.05 one tailed^a^	1.03
Baker et al., 2013	16	S:8	18–21	3 per wk for 8 wks	SP	1 (6)	6RM	S	412.6 ± 71.5 vs. 527.2 ± 74.5	27.8	≤0.05 one tailed^a^	1.57
		M3:8			SP	3 (6)		M3	418.5 ± 49.0 vs. 510.6 ± 62.7	22.0	≤0.05 one tailed^a^	1.64
Kramer et al., 1997	43	S:16	20.3 ± 1.9 [SEM]	3 per wk for 14 wks	Sq	1 (8–12)	8–12RM	S	101.9 ± 20.6 vs. 114.1 ± 18.7	12.0	/	0.62
		M3:14			Sq	3 (8–12)		M3	98.5 ± 27.7 vs. 123.7 ± 43.2	25.6	≤0.05^a^	0.69
		MSV3: 13			Sq	1–3(3–10)		MSV3	111.2 ± 25.6 vs. 135.7 ± 20.6	22.03	≤0.05^a^	1.05
Schlumberger et al., 2001	27	Con: 9	20–40	2 per wk for 6 wks	BP	Con (0)	6RM	Con	28.1 ± 2.4 vs. 27.2 ± 2.9	3.2	/	−0.34
		S:9			BP	1 (6–9)		S	31.7 ± 9.0 vs. 33.0 ± 9.3	4.1		0.14
		M3:9			BP	3 (6–9)		M3	26.9 ± 3.5 vs. 29.7 ± 4.6	10.4	≤0.05^a^	0.69
Humburg et al., 2007	29	Con: 7	23.1–27.1	3 per wk for 9 wks	BP	Con (0)	8–12RM	Con	47.5 ± 15.7 vs. 48.2 ± 17.1	1.47	/	0.04
		S: 22			BP	1 (8–12)		S	56.1± 20.6 vs. 61.7 ± 21.7	10.0	≤0.05^a^	0.26
		M3: 22			BP	3 (8–12)		M3	54.9 ± 21.6 vs. 63.0 ± 23.0	14.8	≤0.05^a^	0.36
Humburg et al., 2007	29	Con: 7	23.1–27.1	3 per wk for 9 wks	LP right leg	Con (0)	8–12RM	Con	155.7 ± 23.1 vs. 149.8 ± 26.2	−3.80	/	−0.24
		S: 22			LP right leg	1 (8–12)		S	174.4 ± 44.7 vs. 188.1 ± 36.4	7.9	≤0.05^a^	0.34
		M3: 22			LP right leg	3 (8–12)		M3	172.7 ± 38.4 vs. 195.3 ± 44.7	13.1	≤0.05^a^	0.54
Humburg et al., 2007	29	Con: 7	23.1–27.1	3 per wk for 9 wks	LP left leg	Con (0)	8–12RM	Con	156.5 ± 31.5 vs. 149.7 ± 33.8	−4.35	/	−0.21
		S: 22			LP left leg	1 (8–12)		S	169.5 ± 40.2 vs. 183.1± 36.1	8.0	≤0.05^a^	0.36
		M3: 22			LP left leg	3 (8–12)		M3	165.4 ± 38.2 vs. 189.9 ± 44.9	14.8	≤0.05^a^	0.59
Radaelli et al., 2014	48	Con: 10	23.5–25.3	3 per wk for 6 months	BP	Con (0)	8–12RM	Con	68.3 ± 11.4 vs. 64.4 ± 8.8	−5.71	/	−0.38
		S:12			BP	1 (8–12)		S	64.5 ± 9.5 vs. 73.2 ± 9.9	13.5	≤0.05^a^, ^c^	0.90
		M3:13			BP	3 (8–12)		M3	73.4 ± 9.4 vs. 86.1 ± 8.4	17.3	≤0.05^a^, ^c^	1.42
		M5:13			BP	5 (8–12)		M5	89.6 ± 9.6 vs. 99.6 ± 5.5	11.2	≤0.05^a^	1.13
Radaelli et al., 2014	48	Con: 10	23.5–25.3	3 per wk for 6 months	LatP	Con (0)	8–12RM	Con	60.5 ± 6.8 vs. 62.2 ± 6.6	2.8	/	0.25
		S:12			LatP	1 (8–12)		S	57.9 ± 10.7 vs. 68.7 ± 9.5	18.7	≤0.05^a^, ^c^	1.07
		M3:13			LatP	3 (8–12)		M3	62.5 ± 6.21 vs. 70.0 ± 4.76	12.0	≤0.05^a^, ^c^	1.36
		M5:13			LatP	5 (8–12)		M5	74.2 ± 9.5 vs. 86.5 ± 6.5	16.6	≤0.05^a^, ^c^	1.51
Radaelli et al., 2014	48	Con: 10	23.5–25.3	3 per wk for 6 months	SP	Con (0)	8–12RM	Con	26.1 ± 7.4 vs. 29.4 ± 7.6	12.6	–	0.44
		S:12			SP	1 (8–12)		S	31.6 ± 7.1 vs. 38.7 ± 9.3	22.5	≤0.05^a^	0.86
		M3:13			SP	3 (8–12)		M3	34.2 ± 7.5 vs. 42.3 ± 6.3	23.7	≤0.05^a^, ^c^	1.17
		M5:13			SP	5 (8–12)		M5	41.5 ± 8.2 vs. 56.1 ± 11.9	35.2	≤0.05^a^, ^c^	1.43
Radaelli et al., 2014	48	Con: 10	23.5–25.3	3 per wk for 6 months	LP	Con (0)	8–12RM	Con	157.8 ± 21.0 vs. 155.0 ± 25.0	−1.8	/	−0.12
		S:12			LP	1 (8–12)		S	170 ± 34.1 vs. 196.7 ± 15.5	15.7	≤0.05^a^	1.01
		M3:13			LP	3 (8–12)		M3	172.5 ± 30.1 vs. 199.2 ± 14.4	15.5	≤0.05^a^, ^c^	1.13
		M5:13			LP	5 (8–12)		M5	178.5 ± 24.4 vs. 201.5 ± 25.4	12.9	≤0.05^a^	0.92

*N, number of subjects; y, years; SD, standard deviation; Reps, repetitions; ES, effect size; S, one-set; M3 three-sets; LP, leg press; RM, repetition maximum; BP, bench press; Sq, squat; SP, shoulder press; MSV3, multiple-sets with changes in training volume; Con, control group; LatP, lateral pull-down; SP, shoulder press; CP, chest press; OP, overhead press*.

a * Significantly greater than prior to training (P ≤ 0.05)*.

b * Significant differences from corresponding groups-exercise values (P ≤ 0.05)*.

c * Significantly greater prior to training (P ≤ 0.01)*.

**Table 8:** Pre- vs. post-strength analysis on single-joint exercise.

**References**	***N***	***N* per group**	**Age (y) [range or mean ± SD]**	**Frequency/duration**	**Testing modality**	**Sets (reps)**	**Training loads**	**Weekly sets per exercise**	**Pre- vs. post [mean ± SD]**	**Pre- vs. post % strength change**	***P*-Value (Pre- vs. post)**	**ES**
Hass et al., 2000	42	S:21	39.2–40.1	3 per wk for 13 wks	LExt	1 (8-12)	8–12RM	S	2.4 ± 0.4 vs. 2.7 ± 0.4	12.5	≤0.05	0.75
		M3:21			LExt	3 (8–12)		M3	2.6 ± 0.4 vs. 2.9 ± 0.4	11.5	≤0.05	0.75
Hass et al., 2000	42	S:21	39.2–40.1	3 per wk for 13 wks	LCurl	1 (8–12)	8–12RM	S	2.0 ± 0.3 vs. 2.1 ± 0.2	5	≤0.05	0.39
		M3:21			LCurl	3 (8–12)		M3	2.1± 0.2 vs. 2.3 ± 0.2	9.5	≤0.05	1.00
Hass et al., 2000	42	S:21	39.2–40.1	3 per wk for 13 wks	BC	1 (8–12)	8–12RM	S	1.0 ± 0.3 vs. 1.1 ± 0.3	10	≤0.05	0.33
		M3:21			BC	3 (8–12)		M3	1.1± 0.3 vs. 1.2 ± 0.3	9.1	≤0.05	0.33
Paulsen et al., 2003	18	S:10	20–30	3 per wk for 6 wks	KExt	1 (7)	7RM	S	125.8 ± 52.8 vs. 144 ± 45.5	14.5	≤0.01^a^	0.37
		M3:8			KExt	3 (7)		M3	117.8 ± 38.2 vs. 142.5 ± 25.2	21.0	≤0.01^a^/≤0.05^b^	0.76
Paulsen et al., 2003	18	S:10	20–30	3 per wk for 6 wks	LCurl	1 (7)	7RM	S	57.3 ± 30.4 vs. 64.8 ± 24.03	13.1	≤0.01^a^	0.27
		M3:8			LCurl	3 (7)		M3	55.9 ± 29.1 vs. 65.3 ± 37.1	16.8	≤0.01^a^/≤0.05^b^	0.25
Kelly et al., 2007	40	Con: 8	22.2–25.3	2 per wk for 8 wks	Con	Con (0)	8RM	Con	135.7 ± 77.1 vs. 127.1 ± 64.6	6.3	/	−0.12
		S:14			KExt	1 (8)		S	163.5 ± 56.4 vs. 171.2 ± 70	4.7	≤0.05	0.12
		M3:18			KExt	3 (8)		M3	171.4 ± 62.0 vs. 200.8 ± 111.1	17.2	≤0.05	0.33
Bottaro et al., 2009	24	S:13	22.2 ± 3.2	2 per wk for 12 wks	KExt	1 (8–12)	8–12RM	S	24.3 ± 3.0 vs. 25.3 ± 2.9	4.1	?	0.34
		M3:11			KExt	3 (8–12)		M3	20.9 ± 3.2 vs. 23.4 ± 2.3	12.0	≤0.05^c^	0.90
Bottaro et al., 2009	24	S:13	22.2 ± 3.2	2 per wk for 12 wks	EExt	1 (8–12)	8–12RM	S	51.4 ± 10.9 vs. 55.2 ± 10.2	7.4	≤0.05^c^	0.36
		M3:11			EExt	3 (8–12)		M3	45.6 ± 5.9 vs. 48.3 ± 8.2	5.9	≤0.05^c^	0.38
Baker et al., 2013	16	S:8	18–21	3 per wk for 8 wks	BC	1 (6)	6RM	S	402.8 ± 54.8 vs. 485.1 ± 48	20.4	≤0.05 one tailed^c^	1.60
		M3:8			BC	3 (6)		M3	421.4 ± 44.1 vs. 499.8 ± 77.4	18.6	≤0.05 one tailed^c^	1.24
Sooneste et al., 2013	8	S:8	25.0 ± 2.1	2 per wk for 12 wks	SPC	1 (8)	8RM	S	9.1 ± 1.6 vs. 10.9 ± 2.5	19.8	≤0.05^c^	0.86
		M3:8			SPC	3 (8)		M3	9.1 ± 1.6 vs. 11.9 ± 2.9	30.8	≤0.05^c^	1.20
Reid et al., 1987	34	S:9	18–35	3 per wk for 8 wks	KFlex	1 (10–12)	6–12RM	S	34.2 ± 6.4 vs. 39.7 ± 8	16.1	≤0.05^c^	0.76
		M3:9			KFlex	3 (6)		M3	35.2 ± 5.3 vs. 40 ± 5.6	13.6	≤0.01^a^	0.88
Reid et al., 1987	34	S:9	18–35	3 per wk for 8 wks	KExt	1 (10–12)	6–12RM	S	80.5 ± 15.8 vs. 95.5 ± 17.8	18.6	≤0.01^a^	0.89
		M3:9			KExt	3 (6)		M3	90.0 ± 16.7 vs. 103.6 ± 16.4	15.1	≤0.01^a^	0.82
Reid et al., 1987	34	S:9	18–35	3 per wk for 8 wks	EFlex	1 (10–12)	6–12RM	S	39.3 ± 4.2 vs. 43.9 ± 6.3	11.7	≤0.01^a^	0.86
		M3:9			EFlex	3 (6)		M3	42 ± 5.2 vs. 45.5 ± 6.9	8.3	≤0.05^c^	0.57
Reid et al., 1987	34	S:9	18–35	3 per wk for 8 wks	EExt	1 (10–12)	6–12RM	S	28.5 ± 7 vs. 35 ± 10.8	22.8	≤0.01^a^	0.71
		M3:9			EExt	3 (6)		M3	33.4 ± 8.1 vs. 40.3 ± 10.3	20.7	?	0.74
Reid et al., 1987	34	S:9	18–35	3 per wk for 8 wks	SFlex	1 (10–12)	6–12RM	S	47.3 ± 10.7 vs. 58.3 ± 10.7	23.3	≤0.01^a^	1.03
		M3:9			SFlex	3 (6)		M3	52.9 ± 11.9 vs. 64.4 ± 9.8	21.7	≤0.05^c^	1.05
Reid et al., 1987	34	S:9	18–35	3 per wk for 8 wks	SExt	1 (10–12)	6–12RM	S	48.2 ± 11.1 vs. 54.8 ± 11.2	13.7	?	0.59
		M3:9			SExt	3 (6)		M3	51.8 ± 9.1 vs. 66.5 ± 11.1	28.4	≤0.01^a^	1.44
Schlumberger et al., 2001	27	Con: 9	20–40	2 per wk for 6 wks	LExt	Con (0)	6RM	Con	44.1 ± 7.7 vs. 44.0 ± 8.6	−0.23	/	−0.01
		S:9			LExt	1 (6–9)		S	44.8 ± 6.8 vs. 47.8 ± 7.9	6.7	≤0.05	0.41
		M3:9			LExt	3 (6–9)		M3	43.7 ± 6.1 vs. 50.6 ± 7.6	15.8	≤0.05	1.00
Humburg et al., 2007	29	Con: 7	23.1–27.1	3 per wk for 9 wks	BC	Con (0)	8–12RM	Con	25.9 ± 11.9 vs. 25.6 ± 12.1	−1.2	/	−0.02
		S: 22			BC	1 (8–12)		S	28.1 ± 9.4 vs. 30.0 ± 9.4	6.8	≤0.05	0.20
		M3: 22			BC	3 (8-12)		M3	26.4 ± 9.5 vs. 30.6 ± 9.4	15.9	≤0.05	0.44

*N, number of subjects; y, years; SD, standard deviation; Reps, repetitions; ES, effect size; S, one-set; M3 = three-sets; per week, number of days trained per week; LExt, leg extension; RM, repetition maximum; LCurl, leg curl; BC, bicep curl; KExt, knee extension; Con, control group; EExt, elbow extension; SPC, seated preacher curl; EFlex, elbow flexion; SFlex, shoulder flexion; SExt, shoulder extension*.

a * Significantly greater than prior to training (P ≤ 0.05)*.

b * Significant differences from corresponding groups-exercise values (P ≤ 0.05)*.

c * Significantly greater than prior to training (P ≤ 0.01)*.

### Untrained Subjects' Effects of Single -vs. Three-Sets on Combined Exercises

Examination of the effects of pre- vs. post-training strength (untrained subjects only) categorized as either S or M3 from six studies (Reid et al., 1987; Paulsen et al., 2003; Humburg et al., 2007; Bottaro et al., 2009; Sooneste et al., 2013; Radaelli et al., 2014). The random-effects model exposed a considerable amount of variability between studies. Q heterogeneity test was: Chi^2^ = 78.30, d.f. = 5, *P* < 0.00001. The heterogeneity statistic *I*^2^ (%) = 94 [interpreted as high, (Higgins et al., 2003)], and the tau^2^ test (between-trials variance) = 0.94. A large effect was observed on untrained only subjects for multi-joint and single-joint exercises combined (ES 1.20; 95% CI 0.39–2.01). Pre- to post-intervention strength change was greater when M3 was compared to S (ES difference 0.22) with statistical significance (*P* = 0.004). The mean for S was 0.60 (95% CI 0.34–0.85). The mean ES for M3 was 0.82 (95% CI 0.52–1.11).

### Trained Subjects' Effects of Single -vs. Three-Sets on Combined Exercises

Separate subgroup examination on the effects of pre- vs. post-training strength (trained subjects only) categorized as either S or M3 from six studies (Kramer et al., 1997; Hass et al., 2000; Schlumberger et al., 2001; Rhea et al., 2002a; Kelly et al., 2007; Baker et al., 2013). The random-effects model exposed a considerable amount of variability between studies. Q heterogeneity test was: Chi^2^ = 29.21, d.f. = 5, *P* < 0.0001. The heterogeneity statistic *I*^2^ (%) = 83 (interpreted as high; Higgins et al., 2003), and the tau^2^ test (between-trials variance) = 0.42. A moderate effect was observed on trained only subjects for multi-joint and single-joint exercises combined (ES 0.63; 95% CI 0.05–1.22). Pre- to post-intervention strength gain was greater when M3 was compared to S (ES difference 0.28) with statistical significance (*P* = 0.03). The mean for S was 0.68 (95% CI 0.35–1.01). The mean ES for M3 was 0.96 (95% CI 0.61–1.31).

### Effects of 1-vs. 3-Sets on Upper Body Exercise

**Figure 3:**
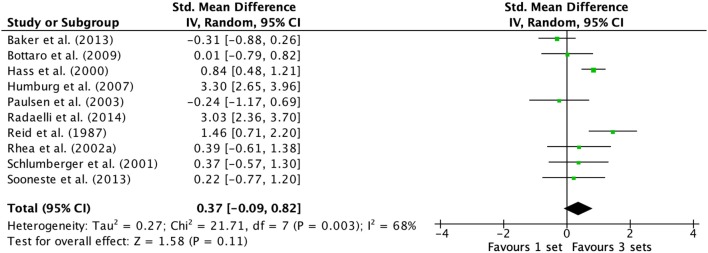
Forest plot of 1 vs. 3-sets (trained and untrained subjects [upper body exercise] combined) with Humburg et al. (2007) and Radaelli et al. (2014) datum excluded.

Examination of upper body exercises (multi-joint and single-joint exercises combined) comprised 10 studies (Reid et al., 1987; Hass et al., 2000; Schlumberger et al., 2001; Rhea et al., 2002a; Paulsen et al., 2003; Humburg et al., 2007; Bottaro et al., 2009; Baker et al., 2013; Sooneste et al., 2013; Radaelli et al., 2014) are displayed in the forest plot (Figure 3). The random-effects model exposed a large amount of variability between studies (*I*^2^ = 93%). Removal of Humburg et al. (2007) and Radaelli et al. (2014) data, resulted in moderate heterogeneity (Q heterogeneity test was: Chi^2^ = 21.71, d.f. = 7, *P* = 0.003). The heterogeneity statistic *I*^2^ (%) = 68 (interpreted as moderate heterogeneity, Higgins et al., 2003), and the tau^2^ test (between-trials variance) = 0.27, and a small effect was observed (ES 0.37; 95% CI −0.09 to 0.82). Pre- to post-intervention strength change was greater when M3 was compared with S (ES difference 0.19) with no statistical significance (*P* = 0.11). The mean ES for S was 0.68 (95% CI 0.42–0.94). The mean ES for M3 was 0.87 (95% CI 0.61–1.14).

### Untrained Subjects' Effects of 1- vs. 3-Sets on Upper Body Exercise

Examination of upper body exercises (multi-joint and single-joint exercises combined) on untrained subjects comprised of six studies (Reid et al., 1987; Paulsen et al., 2003; Humburg et al., 2007; Bottaro et al., 2009; Sooneste et al., 2013; Radaelli et al., 2014). The random-effects model exposed a large amount of variability between studies (*I*^2^ = 94%). Removal of Humburg et al. (2007) and Radaelli et al. (2014) data, resulted in large heterogeneity (Q heterogeneity test was: Chi^2^ = 11.45, d.f. = 3, *P* = 0.010). The heterogeneity statistic *I*^2^ (%) = 74 (interpreted as high heterogeneity, Higgins et al., 2003), and the tau^2^ test (between-trials variance) = 0.54, and a small effect was observed (ES 0.35; 95% CI −0.49 to 1.19). Pre- to post-intervention strength change was greater when M3 was compared with S (ES difference 0.23) with no statistical significance (*P* = 0.42). The mean ES for S was 0.81 (95% CI 0.67–0.95). The mean ES for M3 was 1.04 (95% CI 0.66–1.41). Subgroup examination of S vs. M3 pre- to post-intervention strength differences on trained only and untrained only subjects was not viable due to inadequate study data.

### Trained Subjects' Effects of 1- vs. 3-Sets on Upper Body Exercise

Examination of upper body exercises (multi-joint and single-joint exercises combined) on trained subjects comprised of four studies (Hass et al., 2000; Schlumberger et al., 2001; Rhea et al., 2002a; Baker et al., 2013). Q heterogeneity test was: Chi^2^ = 3.09, d.f. = 3, *P* = 0.38. The random-effects model exposed a low amount of variability between studies. The heterogeneity statistic *I*^2^ (%) = 3 (interpreted as low, Higgins et al., 2003), and the tau^2^ test (between-trials variance) = 0.00. When a random effect analysis was implemented, a moderate effect was detected on trained only subjects for upper body exercise resistance exercises (ES 0.63; 95% CI 0.34–0.92). Pre- to post-intervention strength change was greater when M3 was compared to S (ES difference 0.33) with statistical significance (*P* < 0.0001). The mean for S was 0.59 (95% CI 0.16–1.02). The mean ES for M3 was 0.92 (95% CI 0.53–1.31).

### Effects of 1- vs. 3-Sets on Lower Body Exercise

**Figure 4:**
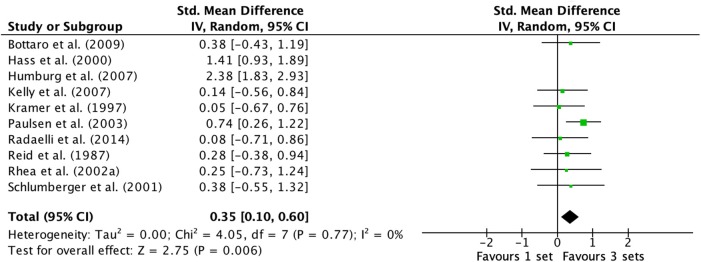
Forest plot of 1 vs. 3-sets (untrained and trained subjects [lower body exercise] combined) with Hass et al. (2000) and Humburg et al. (2007) datum excluded.

Examination of lower body exercises (multi-joint and single-joint exercises combined) on untrained and trained subjects comprised of 10 studies (Reid et al., 1987; Hass et al., 2000; Schlumberger et al., 2001; Rhea et al., 2002a; Paulsen et al., 2003; Humburg et al., 2007; Kelly et al., 2007; Bottaro et al., 2009; Radaelli et al., 2014) are displayed in the forest plot (Figure 4). Q heterogeneity test was: Chi^2^ = 54.00, d.f. = 9, *P* < 0.00001. The random-effects model exposed a high amount of variability between studies *I*^2^ (%) = 83. Removal of Hass et al. (2000) and Humburg et al. (2007) data, resulted in no heterogeneity (Q heterogeneity test was: Chi^2^ = 4.05, d.f. = 7, *P* = 0.77). The heterogeneity statistic *I*^2^ (%) = 0 (interpreted as no heterogeneity, Higgins et al., 2003), and the tau^2^ test (between-trials variance) = 0.00, and a small effect was observed (ES 0.35; 95% CI 0.10–0.60). Pre- to post-intervention strength change was greater when M3 was compared with S (ES difference 0.27) with statistical significance (*P* = 0.006). The mean ES for S was 0.60 (95% CI 0.36–0.84). The mean ES for M3 was 0.87 (95% CI 0.57–1.17).

### Untrained Subjects' Effects of 1- vs. 3-Sets on Lower Body Exercise

Examination of lower body exercises (multi-joint and single-joint exercises combined) on untrained comprised of five studies (Reid et al., 1987; Paulsen et al., 2003; Humburg et al., 2007; Bottaro et al., 2009; Radaelli et al., 2014). Q heterogeneity test was: Chi^2^ = 34.99, d.f. = 4, *P* = < 0.00001). The random-effects model exposed a high amount of variability between studies *I*^2^ (%) = 89. Removal of Humburg et al. (2007) data, resulted in no heterogeneity (Q heterogeneity test was: Chi^2^ = 2.36, d.f. = 3, *P* = 0.50). The heterogeneity statistic *I*^2^ (%) = 0 (interpreted as no heterogeneity, Higgins et al., 2003), and the tau^2^ test (between-trials variance) = 0.00, and a moderate effect was observed (ES 0.49; 95% CI 0.14–0.83). Pre- to post-intervention strength change was greater when M3 was compared with S (ES difference 0.28) with statistical significance (*P* = 0.005). The mean ES for S was 0.75 (95% CI 0.30–1.2). The mean ES for M3 was 1.03 (95% CI 0.51–1.55).

### Trained Subjects' Effects of 1- vs. 3-Sets on Lower Body Exercise

Examination of lower body exercises (multi-joint and single-joint exercises combined) on trained subjects comprised of five studies (Kramer et al., 1997; Hass et al., 2000; Schlumberger et al., 2001; Rhea et al., 2002a; Kelly et al., 2007). Q heterogeneity test was: Chi^2^ = 15.24, d.f. = 4, *P* = < 0.004. The random-effects model exposed a high amount of variability between studies *I*^2^ (%) = 74. Removal of Hass et al. (2000) data, resulted in no heterogeneity (Q heterogeneity test was: Chi^2^ = 0.34, d.f. = 3, *P*-value = 0.95). The heterogeneity statistic *I*^2^ (%) = 0 (interpreted as no heterogeneity, Higgins et al., 2003), and the tau^2^ test (between-trials variance) = 0.00, and a trivial effect was observed (ES 0.18; 95% CI −0.23 to 0.58). Pre- to post-intervention strength change was greater when M3 was compared with S (ES difference 0.32) with no statistical significance (*P* = 0.39). The mean ES for S was 0.63 (95% CI 0.18–1.08). The mean ES for M3 was 0.95 (95% CI 0.43–1.47).

### Effects of 1- vs. 3-Sets on Multi-Joint Only Exercise

**Figure 5:**
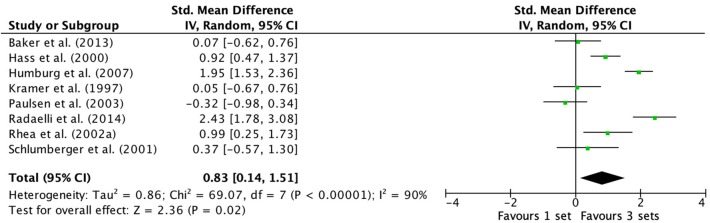
Forest plot of 1 vs. 3-sets (trained and untrained [multi-joint exercise] combined).

Outcomes for S vs. M3 on multi-joint exercise (combined trained and untrained subjects) classified as S or M3 are displayed in the forest plot (Figure 5). The forest plot includes the mean ES and CIs for strength change separated for interventions featuring S and M3 and the overall effect test and heterogeneity analysis. The pooled mean ES estimates of S vs. M3 on multi-joint exercise data comprised of 34 treatment groups from eight studies (Kramer et al., 1997; Hass et al., 2000; Schlumberger et al., 2001; Rhea et al., 2002a; Paulsen et al., 2003; Humburg et al., 2007; Baker et al., 2013; Radaelli et al., 2014). The random-effects model exposed a considerable amount of variability between studies. Q heterogeneity test was: Chi^2^ = 69.07, d.f. = 7, *P* c< 0.00001. The heterogeneity statistic *I*^2^ (%) = 90 (interpreted as high, Higgins et al., 2003), and the tau^2^ test (between-trials variance) = 0.86. A moderate effect was observed for multi-joint exercise and M3 (ES 0.83; 95% CI 0.14–1.51). Pre- to post-intervention strength change was greater when M3 was compared to S (ES difference 0.22) with statistical significance (*P* = 0.02). The mean for S was 0.61 (95% CI 0.34–0.88). The mean ES for M3 was 0.83 (95% CI 0.53–1.13).

### Trained Subjects' Effects of 1- vs. 3-Sets on Multi-Joint Only Exercise

Separate subgroup examination on the effects of pre- vs. post-training strength (trained subjects only) categorized as either S or M3 from five studies (Kramer et al., 1997; Hass et al., 2000; Schlumberger et al., 2001; Rhea et al., 2002a; Baker et al., 2013). The random-effects model exposed a low degree of variability between studies. Q heterogeneity test was: Chi^2^ = 7.73, d.f. = 4, *P* = 0.10. The heterogeneity statistic *I*^2^ (%) = 48 (interpreted as low-moderate, Higgins et al., 2003), and the tau^2^ test (between-trials variance) = 0.07. A moderate effect was observed on trained only subjects for multi-joint exercises combined (ES 0.52; 95% CI 0.10–0.94). Pre- to post-intervention strength change was greater when M3 was compared to S (ES difference 0.25) with statistical significance (*P* = 0.02). The mean for S was 0.67 (95% CI 0.30 to 1.04). The mean ES for M3 was 0.92 (95% CI 0.56–1.28). Subgroup analysis of S vs. M3 pre-to post-intervention strength differences on untrained subjects was not feasible due to inadequate study data.

### Effects of 1- vs. 3-Sets on Multi-Joint Upper Body Exercise

Subgroup analysis on the effects of pre- vs. post-training strength multi-joint upper body exercises (trained and untrained subjects) categorized as either S or M3 from seven studies (Hass et al., 2000; Schlumberger et al., 2001; Rhea et al., 2002a; Paulsen et al., 2003; Humburg et al., 2007; Baker et al., 2013; Radaelli et al., 2014). The random-effects model exposed a significant amount of variability between studies. Q heterogeneity test was: Chi^2^ = 70.88, d.f. = 6, *P* < 0.00001. The heterogeneity statistic *I*^2^ (%) = 92 [interpreted as high, (Higgins et al., 2003)] and the tau^2^ test (between-trials variance) = 1.57. A large effect was observed (ES 1.15; 95% CI 0.17–2.12). Pre- to post-intervention strength change was greater when M3 was compared to S (ES difference 0.24) with statistical significance between RT set volumes (*P* = 0.02). The mean for S was 0.55 (95% CI 0.23–0.86). The mean ES for M3 was 0.79 (95% CI 0.44–1.14).

### Trained Subjects' Effects of 1– vs. 3-Sets on Multi-Joint Upper Body Exercise

Subgroup examination of S vs. M3 pre- vs. post-training strength differences on trained subjects multi-joint upper body exercises comprised of four studies (Hass et al., 2000; Schlumberger et al., 2001; Rhea et al., 2002a; Baker et al., 2013). The random-effects model exposed a low to moderate amount of variability between studies. Q heterogeneity test was: Chi^2^ = 4.56, d.f. = 3, *P* = 0.21. The heterogeneity statistic *I*^2^ (%) = 34 (interpreted as low-moderate, Higgins et al., 2003), and the tau^2^ test (between-trials variance) = 0.07. A moderate effect was observed (ES 0.52; 95% CI 0.08–0.96). Pre- to post-intervention strength gain was greater when M3 was compared to S (ES difference 0.32) with statistical significance (*P* = 0.02). The mean for S was 0.59 (95% CI 0.09–1.08). The mean ES for M3 was 0.91 (95% CI 0.49–1.32). Subgroup examination of S vs. M3 pre- to post-intervention strength differences on untrained subjects was not feasible due to limited study data.

### Effects of 1- vs. 3-Sets on Multi-Joint Lower Body Exercise

Analysis of S vs. M3 pre- to post-intervention strength differences on subject's multi-joint lower body exercises comprised of five studies (Kramer et al., 1997; Rhea et al., 2002a; Paulsen et al., 2003; Humburg et al., 2007; Radaelli et al., 2014). The random-effects model showed a significant amount of variability between studies. Removal of (Humburg et al., 2007) data resulted in no heterogeneity (Q heterogeneity test was: Chi^2^ = 0.13, d.f. = 3, *P* = 0.99). The heterogeneity statistic *I*^2^ (%) = 0 (interpreted as none, Higgins et al., 2003), and the tau^2^ test (between-trials variance) = 0.00 and trivial effect observed (ES 0.09; 95% CI −0.32 to 0.51). Pre- to post-intervention strength change was greater with M3 compared with S (ES difference 0.17) with no statistical significance (*P* = 0.66). The mean ES for S was 0.81 (95% CI 0.41–1.21). The mean ES for M3 was 0.98 (95% CI 0.45–1.51). Subgroup examination of S vs. M3 pre- to post-intervention strength differences on trained only and untrained only subjects was not feasible due to inadequate study data.

### Effects of 1- vs. 3-Sets on Single-Joint Exercise

**Figure 6:**
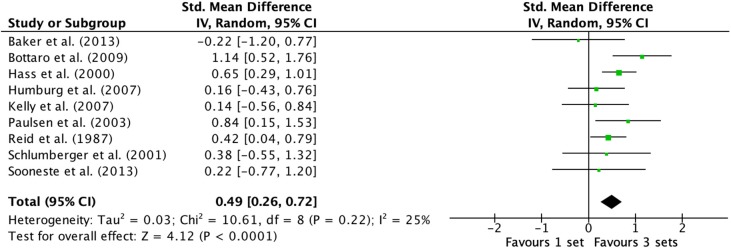
Forest plot of 1 vs. 3-sets (trained and untrained [single-joint exercises] combined).

Outcomes for 1-vs.-3 sets categorized as S or M3 for single-joint resistance exercises are displayed in the forest plot (Figure 6). The pooled mean ES estimates of single-joint resistance exercises comprised of 36 treatment groups from nine studies (Reid et al., 1987; Hass et al., 2000; Schlumberger et al., 2001; Paulsen et al., 2003; Humburg et al., 2007; Kelly et al., 2007; Bottaro et al., 2009; Baker et al., 2013; Sooneste et al., 2013). The random-effects model exposed a low to moderate amount of variability between studies. Q heterogeneity test was: Chi^2^ = 10.61, d.f. = 8, *P* = 0.22. The heterogeneity statistic *I*^2^ (%) = 25 (interpreted as low-moderate, Higgins et al., 2003), and the tau^2^ test (between-trials variance) = 0.03 and a small effect was observed (ES 0.49; 95% CI 0.26–0.72). Pre- to post-intervention strength change was marginally greater with M3 compared with S (ES difference 0.19) with statistical significance (*P* < 0.0001). The mean ES for S was 0.57 (95% CI 0.26–0.87). The mean ES for M3 was 0.76 (95% CI 0.54–0.98).

### Trained Subjects' Effects of 1- vs. 3-Sets on Single-Joint Exercise

Subgroup examination on the effects of pre- vs. post-training strength (trained subjects only) categorized as either S or M3 from four studies (Hass et al., 2000; Schlumberger et al., 2001; Kelly et al., 2007; Baker et al., 2013). Q heterogeneity test was: Chi^2^ = 3.91, d.f. = 3, *P* = 0.27. The random-effects model exposed a low amount of variability between studies. The heterogeneity statistic *I*^2^ (%) = 23 (interpreted as low, Higgins et al., 2003), and the tau^2^ test (between-trials variance) = 0.04. When a random effect analysis was implemented, a small effect was detected on trained only subjects for single-joint resistance exercises (ES 0.37; 95% CI −0.01 to 0.75). Pre- to post-intervention strength change was greater when M3 was compared to S (ES difference 0.09) with no statistical significance (*P*-value = 0.06). The mean for S was 0.75 (95% CI 0.12–1.28). The mean ES for M3 was 0.84 (95% CI 0.39–1.28).

### Untrained Subjects' Effects of 1- vs. 3-Sets on Single-Joint Exercise

Examination of the effects of pre- vs. post-training strength (untrained subjects only) categorized as either S or M3 from five studies (Reid et al., 1987; Paulsen et al., 2003; Humburg et al., 2007; Bottaro et al., 2009; Sooneste et al., 2013). The random-effects model showed a low to moderate amount of variability between studies. Q heterogeneity test was: Chi^2^ = 6.76, d.f. = 4, *P* = 0.15. The heterogeneity statistic *I*^2^ (%) = 41 (interpreted as low-moderate, Higgins et al., 2003), and the tau^2^ test (between-trials variance) = 0.06, and a moderate effect was observed on untrained only subjects for single-joint exercises (ES 0.56; 95% CI 0.21–0.91). Pre- to post-intervention strength change was marginally greater when M3 was compared to S (ES difference 0.23) with statistical significance (*P* = 0.002). The mean for S was 0.51 (95% CI 0.24–0.77). The mean ES for M3 was 0.74 (95% CI 0.47–1.02).

### Effects of 1- vs. 3-Sets on Upper Body Single-Joint Exercise

Subgroup examination of upper body single-joint exercises are presented in the forest plot. The pooled mean ES estimates comprised of six studies (Reid et al., 1987; Hass et al., 2000; Humburg et al., 2007; Bottaro et al., 2009; Baker et al., 2013; Sooneste et al., 2013). The random-effects model exposed a low to moderate amount of variability between studies. Q heterogeneity test was: Chi^2^ = 2.74, d.f. = 5, *P* = 0.74. The heterogeneity statistic *I*^2^ (%) = 0 (interpreted as no heterogeneity, Higgins et al., 2003), and the tau^2^ test (between-trials variance) = 0.00, and a trivial effect was detected (ES 0.20; 95% CI −0.07 to 0.47). Pre- to post-intervention strength change was comparable when M3 was compared with S (ES difference 0.07) with no statistically significance (*P* = 0.16). The mean ES for S was 0.69 (95% CI 0.28–1.11). The mean ES for M3 was 0.76 (95% CI 0.42–1.09). Subgroup examination of S vs. M3 pre- to post-intervention strength differences on trained only and untrained only subjects was not possible due to inadequate study data.

### Effects of One –vs. Three-Sets on Lower Body Single-Joint Exercise

Examination on lower body single-joint exercises comprised of six studies (Reid et al., 1987; Hass et al., 2000; Schlumberger et al., 2001; Paulsen et al., 2003; Kelly et al., 2007; Bottaro et al., 2009). The random-effects model exposed a moderate amount of variability between studies (*I*^2^ = 63%). Removal of Hass et al. (2000) data, resulted in no heterogeneity (Q heterogeneity test was: Chi^2^ = 2.24, d.f. = 4, *P* = 0.69. The heterogeneity statistic *I*^2^ (%) = 0 (interpreted as no heterogeneity, Higgins et al., 2003), and the tau^2^ test (between-trials variance) = 0.00, and a small effect was observed (ES 0.41; 95% CI 0.08–0.74). Pre- to post-intervention strength change was greater when M3 was compared with S (ES difference 0.32) with statistical significance (*P* = 0.02). The mean ES for S was 0.40 (95% CI 0.17–0.63). The mean ES for M3 was 0.72 (95% CI 0.51–0.93). Subgroup examination of S vs. M3 pre- to post-intervention strength differences on trained only and untrained only subjects was not viable due to inadequate study data.

## Discussion

This paper is the first meta-analytical review that compares terrestrial RT set-volume evidence and provides recommendations for pre-spaceflight preparation. This meta-analysis sought to determine whether M3 is associated with more significant strength gains than S per resistance exercise in RT programmes (Table 9). Additionally, results from this meta-analysis indicate the future need for research that explores appropriate physical RT implemented during spaceflight preparation. When trained and untrained subjects were combined, M3 was associated with greater ES in both combined multi-joint and single-joint exercises compared to S (Table 9). However, significant heterogeneity existed when combining different population groups (trained and untrained) and exercise type (multi-joint and single-joint). When body segmentation was analyzed (upper and lower body) M3 reported greater ES compared to S. Subgroup analysis on upper body only (trained and untrained combined and untrained studies only) reported marginally greater gains in strength when M3 was compared to S but were not significant. Trained subjects only reported statistically significant pre-post-strength changes (*P* < 0.0001) in upper body RT exercise. The data for lower body exercise reported that M3 was associated with greater ES in both combined population group (*P* = 0.02) and untrained subjects only (*P* = 0.02), but not trained subjects (*P* = 0.39).

**Table 9:** Summary of main effects of 1 vs. 3-sets and strength change.

**Main effects**	**ES mean**	**95% CI**	***I*^**2**^(%)**	**ES difference S vs. M3**	***P-*value**
**COMBINED MULTI-JOINT AND SINGLE-JOINT EXERCISE**					
Trained and untrained combined	0.93	0.41–1.45	92	0.25	0.0005^a^
Untrained subjects only	1.20	0.39–2.01	94	0.22	0.004^a^
Trained subjects only	0.63	0.05–1.22	83	0.28	0.03^a^
**UPPER BODY EXERCISE (MULTI-JOINT AND SINGLE-JOINT COMBINED)**					
Trained and untrained combined	0.37	0.09–0.82	68	0.19	0.11
Untrained subjects only	0.35	−0.49–1.19	74	0.23	0.42
Trained subjects only	0.63	0.34–0.92	3	0.33	<0.0001^a^
**LOWER BODY EXERCISE (MULTI-JOINT AND SINGLE-JOINT COMBINED)**					
Trained and untrained combined	0.35	0.10–0.60	0	0.27	0.006^a^
Untrained subjects only	0.49	0.14–0.83	0	0.28	0.005^a^
Trained subjects only	0.18	−0.23–0.58	0	0.32	0.39
**MULTI–JOINT ONLY EXERCISE**					
Trained and untrained combined	0.83	0.14–1.51	90	0.22	0.02^a^
Trained subjects only	0.52	0.10–0.94	48	0.31	0.02^a^
**MULTI-JOINT UPPER BODY**					
Trained and untrained combined	1.15	0.17–2.12	92	0.24	0.02^a^
Trained subjects only	0.52	0.08–0.96	34	0.32	0.02^a^
**MULTI-JOINT LOWER BODY**					
Trained and untrained combined	0.09	−0.32–0.51	0	0.17	0.66
**SINGLE-JOINT EXERCISE**					
Trained and untrained combined	0.49	0.26–0.72	25	0.19	<0.0001^a^
Untrained subjects only	0.56	0.21–0.91	41	0.23	0.002^a^
Trained subjects only	0.37	−0.01–0.75	23	0.09	0.06
**UPPER BODY SINGLE-JOINT EXERCISE**					
Trained and untrained combined	0.20	−0.07–0.47	0	0.07	0.16
**LOWER BODY SINGLE-JOINT EXERCISE**					
Trained and untrained combined	0.41	0.08–0.74	0	0.32	0.02^a^

*ES, effect size; 95% CI, 95% confidence level; I^2^, I-squared index test; S, one-set; M3, multiple-sets*;

a * = statistically different between treatments*.

Analysis of combined population groups on pooled multi-joint exercise and upper body multi-joint exercise reported greater strength gains with M3 compared to S. However, multi-joint lower body exercise reported marginally greater gains in strength with M3 compared to S, but these were not statistically significant. Subgroup analysis on untrained subjects only reported greater ESs on single-joint only exercises with M3 compared to S, with moderate heterogeneity detected between studies. When subgroup analysis was performed on trained only subjects on combined multi-joint exercises, upper body multi-joint only, and lower body multi-joint only the ES for M3 was statistically greater compared to S for strength gains. However, analysis of single-joint only exercise on trained subjects cannot fully support the contention that M3 produced greater ESs than S, as findings were not statistically significant. The results of this analysis provide support for the use of additional sets (up to three-sets) per exercise for increasing muscle strength. However, both S and M3 demonstrated significant pre-post-strength differences in multi-joint and single-joint exercises.

### Ground-Based Meta-Analytical Recommendations for Untrained Individuals

The scientific literature on daily set-volume has been heavily contested with some suggesting that S produce similar adaptations to MS (Silvester et al., 1982; Messier and Dill, 1985; Reid et al., 1987; Pollock et al., 1993; Starkey et al., 1996; Hass et al., 2000). However, others indicate that MS produces greater strength, hypertrophy, and power adaptations (Kraemer, 1997; Hass et al., 2000; Borst et al., 2001; Marx et al., 2001; McBride et al., 2003; Paulsen et al., 2003). In the context of pre-flight conditioning, the initial physical training status of each crew member must be fully considered. Specifically, as the inclusion of smaller doses of daily RT (e.g., S per exercise) may be appropriate to develop muscular strength in less conditioned (untrained) crew members, whereas more substantial doses of RT (e.g., ≥M3 per exercise) may be essential to attain further strength.

Currently, the prescription of RT for pre-flight conditioning is recommended from ground-based models and experience gained during previous missions. Present recommendations for ST consist of one-to-three-sets per exercises for untrained individuals and MS used with variations in RT volume and intensity over a period for trained individuals [American College of Sports Medicine Position Stand (ACSM), 2009]. These recommendations on terrestrial RT set-volume have been subject to strong criticism due to reported methodological constraints within the included meta-analyses (Smith and Bruce-Low, 2004; Winett, 2004; Otto and Carpinelli, 2006; Carpinelli, 2012; Fisher, 2012). For example, the meta-analysis by Rhea et al. (2003) presented data that may have nullified the mean ES and spuriously affected the results. This is due to increasing the heterogeneity of the meta-analysis, therefore, erroneously favoring MS programming. Rhea and colleagues reported that untrained subjects should perform four-sets for strength increases (ES = 2.28 ± 1.96 SD), compared with S (ES = 1.16 ± 1.59 SD), two-sets (ES = 1.75 ± 3.23 SD), and M3 (ES = 1.94 ± 3.23 SD). However, the ES standard deviation for M3 was significantly larger in comparison to the other treatment groups, with no explanation provided. Otto and Carpinelli (2006) stated that several critical errors could invalidate the results including bias in the selection process of studies, incorrect classification of subjects training status, and variances of RT loading.

A meta-analysis by Wolfe et al. (2004) suggested that untrained subjects in the initial stages of training (6–15 weeks) perform with an S programme. Wolfe et al. (2004) also reported that untrained subjects had comparable pre-post-strength gains as those of trained individuals when performing MS. When subjects performed, RT exercises to physical failure vs. subjects' perceived end, multiple-set programmes generated more substantial increases in strength compared to S (*P* ≤ 0.002). A meta-analysis by Krieger (2009) examined the effects of S vs. multiple-sets of exercises on strength and suggested that two-to-three-sets were associated with a more significant ES than S. Krieger (2009) concluded that two-to-three-sets per resistance exercise were associated with 46% greater strength gains than S in both trained and untrained subjects. Finally, a meta-analysis by Fröhlich et al. (2010) of 72 studies found S training regimes to be the equivalent of multiple-set training for short intervention phases but multi-set training to be superior overextended RT intervention periods.

This current meta-analysis provides evidence that supports the contention that M3 RT leads to greater strength gains than S programming for untrained individuals or those astronauts that may be on the early phase of pre-flight conditioning. However, this analysis examined the differences in pre-post-strength gain between body segments and joint types rather than only pooling RT exercises together to generate ES. For upper body exercise, untrained subjects demonstrated a larger pooled mean ES estimate for M3 0.82 (95% CI 0.52–1.11) compared with S (0.60; 95% CI 0.34–0.85), however these results were not statistically significant (*P* = 0.42). Also, a significant degree of heterogeneity was present when exercises were “pooled” together. This intriguingly may explain why other previous meta-analyses reported varying results with regards to specific set-volume and encountered criticism from others as the included studies combined RT exercises to aggregate ES. Subgroup examination on untrained subjects was only feasible for upper and lower body and single-joint only exercises due to limited available data for multi-joint exercise. Untrained subjects pre- to post-strength gains on upper body exercise was greater when M3 was compared with S (ES difference 0.23) but was not statistically significant (*P* = 0.42). In contrast, lower body exercises reported significantly greater strength changes (*P* = 0.005) with M3 compared with S (ES difference 0.28). Strength gains on single-joint exercises exposed a larger pooled mean ES estimate for M3 (0.74; 95% CI 0.47–1.02) compared with S (0.51; 95% CI 0.24–0.77). When S was used as a control group and M3 used as the experimental set-volume, a moderate ES of 0.56 (95% CI 0.21–0.91; *P* = 0.002) suggested that M3 was effective in generating larger strength increases.

Even though evidence from this analysis supports increase set-volume for strength gain, caution is warranted concerning the interpretation of the findings as exercises performed in the included studies may not transfer toward improved operational task functioning. Furthermore, even though superior increases in strength may be produced with M3, the necessary time and physical effort required to attain such strength developments may not be essential for preparation toward spaceflight. The time to perform this additional conditioning may be better served in completing other necessary operational tasks.

### Ground-Based Meta-Analytical Recommendations for Trained Individuals

Current recommendations from NASA for pre-flight conditioning suggest that trained astronauts who are in the final stages of pre-spaceflight preparation perform MS with variations in RT volume and intensity (American College of Sports Medicine Position Stand (ACSM), 2009; Garber et al., 2011). Several ground-based meta-analyses (Rhea et al., 2003; Peterson et al., 2004; Wolfe et al., 2004; Krieger, 2009; Fröhlich et al., 2010) that are included within the American College of Sports Medicine Position Stand (ACSM) (2009) recommendations have reported superior results when performing MS for trained subjects. Rhea et al. (2003) reported strength increases in the bench press of 20% for S, compared with a 33% increase with M3. Pre- to post leg strength increased by 25.4% for S compared with an increase of 52.1% with M3. However, the inclusion of the Rhea et al. (2002a) study may have generated errors or bias when comparing the means of the one-set and three-set groups (bench press ES = 2.3 and leg press ES 6.5). Also, the post-training standard deviation bench press findings were two-to-three times greater to the pre-training standard deviation in both groups, and the authors did not provide confidence intervals for ES. The meta-analysis by Peterson et al. (2004) stated that experienced athletes should complete eight-sets per muscle group to increase muscular strength. However, due to the low number of ES in the eight-set group, these conclusions may be unreliable when evaluating the specific percentage of 1RM training. An additional meta-analysis performed by Wolfe et al. (2004) reported that MS generated more significant increases in strength (*P* ≤ 0.001) for trained subjects.

This current meta-analysis cautiously provides evidence concerning increased sets and strength gain with support toward the use of additional sets (up to M3) compared to S for trained astronauts pre-flight conditioning programme. In this analysis, we reveal that multi-joint only exercises on trained subjects demonstrated a larger pooled mean ES estimate for M3 (0.99; 95% CI 0.60–1.38) compared with S (0.68; 95% CI 0.27–1.10) suggesting that M3 was more effective in producing larger strength gains. Further examination of upper body multi-joint only exercises further supports the use of M3. The pooled mean ES estimate for S was 0.59 (95% CI 0.09–1.08) compared with M3 (0.91; 95% CI 0.49–1.32) suggesting that M3 was more effective in producing strength gains. For single-joint exercises on trained subjects, there was a larger pooled mean ES estimate for M3 (0.84; 95% CI 0.39–1.28) compared with S (0.75; 95% CI 0.12–1.28). However, this was not statistically significant in producing more substantial strength gains.

Exercise prescription currently implemented by space agencies include the prescription of greater RT set-volume, but crew members do not perform the volume in a linear manner. It could be hypothesized, therefore, that by increasing set-volume prior to the commencement of spaceflight may develop astronaut's pre-flight levels of upper and lower body strength. Similarly, Krieger (2010) found that this range of sets and reps was superior in producing muscular hypertrophy. This suggests that the atrophy effects of long duration spaceflight could be mitigated, but not prevented, by a more rigorous deployment of multi-set RT methods. Astronauts that are appropriately conditioned and have progressed in their individualized RT toward a trained state should perform MS per exercise to maximize muscular strength, as it could safeguard against decreases in strength. However, caution should be given with the prescription of daily RT set-volume as exposing astronaut's to chronically high training volume may expose crew members to overtraining syndrome before spaceflight. Also, the inclusion of increased set-volume may also come at the determinant of the application toward training intensity and loading (e.g., 85% 1RM) and may feasibly inhibit strength development (Stone et al., 2006). Collectively, when accounting for other programme variables (RT loading and training frequency), M3 appears to a certain degree to be more advantageous for strength development in individuals that are trained. However, careful consideration must be given toward the astronauts initial pre-training status because of variance between individuals. Furthermore, comprehensive monitoring should be considered to prevent the potential of excess fatigue and to overtrain (Day et al., 2004; Halson, 2014).

### Future Considerations Toward Pre-spaceflight Muscle Conditioning

Historically, exercise has been prescribed as a method to counteract the undesired physiological effects of long duration exposure to weightlessness and to safeguard astronaut's health (both acute and chronic). Space agencies have recently acknowledged that the pre-flight physical fitness of astronauts must be higher than age centered normative measurements before spaceflight. The preparation of crew members has advanced from methods that traditionally only improved physiological tolerance toward spaceflight to practices that prepare individuals to live and work in space (Garshnek, 1989). These physical training protocols are employed as a method of countering detrimental physiological adaptations that result from μG. The physical conditioning and preparation of astronauts for spaceflight are essential; however, space agencies approach mission preparation differently. It is evident that most agencies foster a graded dose-response continuum with the initial phase of training emphasizing lower set-volume progressing to MS at the concluding stages of flight preparation.

Based on evidence generated from this analysis, it would be appropriate to suggest that astronauts perform RT via a graded dose-response continuum in preparation for in space transit. However, published research in this area is limited with evidence centered on ground-based studies from older adult populations and bed rest studies that may not fully replicate the physiological pre-spaceflight muscle conditioning status of astronauts. More evidence is required concerning the effects of RT bouts and the dose-response relationship on the training status, age and sex differences for protecting the muscular systems. A greater understanding of the mechanisms for physiological conditioning would, therefore, help to develop appropriate individual centered countermeasures that mitigate deconditioning and must be an essential area of focus for future space physiology research. Ensuring each astronaut's pre-spaceflight strength is at the desired level may help to counteract in-flight and post-flight physiological stress which is paramount for the development of a viable human space exploration programme that goes beyond Earth's orbit.

### Strengths and Limitations

There are numerous strengths of this meta-analysis that separate it from previous investigations that compare S vs. M3 configurations. Considerations toward evidence derived from previous critical reviews that identify limitations regarding the quality and selection of included studies that may bias outcomes have been applied [in part]. These review articles debated the findings from previous meta-analytical studies, identifying confounding factors and imprecisions that may have influenced outcome reliability. Previous review articles in this area, have reported issues concerning robustness and rigor that control confounding variables when comparing strength outcomes. Subsequently, this meta-analysis has been founded on critical consensus, current studies, and previous evidence gathered from meta-analyses presenting a re-examination of the evidence. Greater emphasis was placed on strict inclusion and exclusion criteria, specifying why each study was excluded to allow for greater transparency. The identification and the inclusion criteria of studies were restricted to reduce heterogeneity between studies making it easier to apply the results to specific population groups (trained and untrained). This enabled direct assessments to be drawn from studies that compared S and M3 while endeavoring to control for other variables that influence strength outcomes.

This meta-analysis endeavored to limit where possible publication bias which is the most significant source of type I error (the increase of false positive results). Graphical representation via funnel plots evaluated the presence of publication bias which was not present in other meta-analytical studies (Rhea et al., 2002b, 2003; Peterson et al., 2004; Wolfe et al., 2004; Krieger, 2010). Where no publication bias was present, the ES of each included study is distributed symmetrically around the underlying true ES, with a more random variation of this value in smaller studies. Asymmetry in the plot is suggestive of bias, most often due to smaller studies which are non-significant or have an effect in the opposite direction from that assumed (Greco et al., 2013). Within our design, we were conscious of potential heterogeneity and implemented a multi-level model as a method for assessing heterogeneity across studies. Also, visual inspection of forests plots further verified this and indicated the need for further subgroupings of studies which reduced the degree of heterogeneity.

Moreover, subgroup analysis was performed to allow for the assessment of training status (trained and untrained) separately and also the analysis of multi-joint and single-joint exercises. This helped to reduce heterogeneity between groups and allowed for more consistent comparisons to be made. Lastly, efforts were made to restrict the subject pool to population groups (trained and untrained) that were physically active and within a comparable age range that mirrored that of an astronaut's pre-flight fitness status. This is due to the disparity existing with astronaut's fundamental physiological characteristics, with physical fitness levels ranging from sedentary individuals who performed little or no physical activity to athletic standard.

Limitations of this meta-analysis include the use of small sections of the published information that is often derived from an insufficient range of methodological designs that has been determined by the inadequacy of available primary data. As with all meta-analyses, limitations and restrictions exist that include the assessment of aggregate outcomes that inevitably do not assess the same construct (i.e., “comparing apples and oranges”). This is the process of search and retrieval of available journal articles, and the potential effect of publication bias (Rosenthal, 1991). Cooper and Hedges (1994) stated that “retrieval bias” may exist even when robust inclusion criteria for gathering journals articles is applied. For example, in this analysis, retrieval bias may have occurred due to the selection and retrieval of journal articles published only in English. Although we strived to include journal articles from high-quality sources, the number of relevant studies was restricted, and there remained variances in experimental design and control among included studies. For example, four of the 12 included journal articles used RCTs. The other eight used a RAN that did not include a control group; instead, a repeated measures design was used with a baseline measure implemented as the control. However, baseline measures were not uniformly applied across those studies.

In this meta-analysis, the strength increases may be due to other physiological factors that have been acknowledged to affect strength changes including sex, age, physical activity levels, prior training history, genetics and endocrine status. This analysis was unable to subcategories subjects into either male or female population groups; instead, it combined population groups. Several studies have reported that sex influences muscle morphology and functioning (Chorney and Bourgeois, 1999; Sale, 1999). Häkkinen et al. (1996) reported that males have greater muscle strength and size than women due to higher levels of anabolic hormones and greater body mass. Also, it has been inferred that due to lower blood androgen levels in women that the response to RT would induce less muscle hypertrophy compared to men. Equally, some studies have reported no differences between sexes with similar improvements in strength adaptations (Colliander and Tesch, 1991; O'Hagan et al., 1995; Roth et al., 2001).

For this analysis, every effort was made to assess strength outcomes of comparable training designs and methodological construct. However, attempting to include studies that had a standardized RT programme was problematic. Even when attempting to control for variance within studies, confounding issues that may have influenced strength development were present. The RT loading had various ranges (63–93% 1RM) that could not be controlled for which could affect strength gains. As training loading is one of the most critical parameters of ST and the volume of RT training makes it challenging to provide a clear dose-response relationship that supports M3 compared to S programming for strength gain. The previous analyses found that loading of 60% of subjects 1RM (Rhea et al., 2003; Peterson et al., 2004) was sufficient to increase strength for untrained subjects, with trained subjects recommended to perform between 80 and 85% of their 1RM [American College of Sports Medicine Position Stand (ACSM), 2009]. The disparity in the training programme type and the order of resistance exercise between groups were not comparable in all included studies which feasibly could impact upon the set number and muscular strength. Scientific literature is currently equivocal regarding the implementation of heavy or light RT loads for muscular adaptations (Fisher et al., 2017). It must be acknowledged that the different repetition ranges included within this meta-analysis may also be a possible limitation. Several studies have reported comparable strength gains when training at different repetition ranges (Morton et al., 2011; Fisher et al., 2017) whereas others have shown improved strength when individuals trained with higher RT load and a low number of repetitions (Campos et al., 2002). This could be explained by the specificity of the strength test, as training with a low number of repetitions is closer to the 1RM test (Buckner et al., 2017; Gentil et al., 2017).

Finally, strength adaptations may have resulted from the performance of repeated 1RM testing as the resistance exercise loading specificity of the 1RM-tested exercises may have indirectly affected the subject's strength. For example, a subject's pre-to-post leg extension results may have increased, due to the performance of the leg press but not to the same amount as a leg press itself. This is supported by Dankel et al. (2016) who performed 1RM and maximum voluntary contraction testing on elbow flexion. The results intimated that the increase in the 1RM result might not be solely correlated to set-volume, but due to the “learning effect” of the specific resistance exercise. This could explain the variation in untrained subjects' strength due to the principle of training specificity, as neurological learning effects could, therefore, explain the broad range of heterogeneity between the inexperienced group.

## Conclusion

The prescription for increasing maximal strength is a multifaceted process that involves careful manipulation of RT programme variables. A known association between sets (training volume) and strength improvement would be valuable to researchers, clinicians and astronauts whether they are pre-or in-flight. Recommendations on the appropriate number of sets per session per RE that elicit strength improvements is a contentious issue. The current academic literature does little to resolve this debate with the sets to strength gain correlation remaining unquantified. It is difficult to fully draw accurate conclusions from previous meta-analyses because of confounding training variables including programme duration, training frequency, muscle groups trained and measured, strength testing procedures, repetition velocity, and training status of the subjects. Independent of training status, M3 protocols should be included when maximal strength development is the primary objective of the training routine. However, single-set programmes also produce considerable increases in muscular strength, albeit not to the same degree as that of M3, and are advocated when training time is restricted or at the start of pre-flight conditioning programme.

For astronauts at the end of pre-flight conditioning and considered to be trained individuals, the use of M3 may be at the minimum necessary if not mandatory for trained individuals to further produce strength gains. These physically trained individuals may benefit from additional time and training volume to develop minor increases in performance customarily observed at this level of training progression. However, attention should be given with these advanced trainees due to the interaction of additional RT volume and time with the other fitness and mission orientated goals. However, it is essential to clarify that these modestly greater strength improvements occur at the expense of additional training effort. Ultimately, M3 entails 200–400% greater training volume than S training modes. For astronauts lacking in time, a reduced number of sets (S) per exercise may be what is needed to achieve their desired training goals. It is essential to consider this issue as a lack of time can be a barrier to exercise adherence, and one should be cautious before recommending M3 per exercise to deconditioned astronauts as it can hinder training progression. Also, astronauts in spaceflight preparation are subject to tight scheduling and most often experience time constraints. In such phases of limited time-availability, S can still provide some (yet less) training benefit and should be favored as an alternative exercise mode rather than nothing at all. More research is necessary to define the dose-response relationship for terrestrial RT. However, due to the unique environmental conditions experienced during spaceflight, it may be difficult to fully translate evidence generated from conventional terrestrial RT intervention studies to μG.

## Author Contributions

All authors listed have made a substantial, direct and intellectual contribution to the work, and approved it for publication.

### Conflict of Interest Statement

PJ is employed by KBR. The remaining authors declare that the research was conducted in the absence of any commercial or financial relationships that could be construed as a potential conflict of interest.

## Abbreviations

ACSM: American College of Sports Medicine
Chi^2^: Chi-square
CI: 95% confidence intervals
d.f.: Degrees of freedom
ES: Effect size
*I*^2^: I-squared index test
ISS: International Space Station
IV: Inverse variance
M: Mean
M3: Three-set
MVC: Maximum voluntary contraction
NASA: National Aeronautics and Space Administration
*n*: Sample size number
*P*: Probability
PEDro: Physiotherapy evidence database
PRISMA: Preferred reporting items for systematic reviews and meta-analyses
Q: Cochran Q
RAN: Randomized trials
RCTs: Randomized control trials
RevMan: Review manager
RT: Resistance training
S: One-set
SD: Standard deviation
SE: Standard error
SEM: Standard error of measurement
SMD: Standardized mean difference
Tau^2^: Tau-square
μG: Microgravity
1RM: One repetition maximum.
